# Supplementary material for: SIEVE: joint inference of single-nucleotide variants and cell phylogeny from single-cell DNA sequencing data
Source: Genome Biol. 2022 Nov 30;23:248. doi: 10.1186/s13059-022-02813-9 (PMC9714196; doi:10.1186/s13059-022-02813-9)
Supplement: Supplementary file 1 — Additional file 1: Supplementary Figs. S1-S21, Tables S1-S4, and Note. [file 13059_2022_2813_MOESM1_ESM.pdf]

SIEVE: joint inference of single-nucleotide variants and cell phylogeny from single-cell DNA sequencing data

Additional file 1

Senbai Kang[1], Nico Borgsmüller[2,3], Monica Valecha[4,5], Jack Kuipers[2,3], Joao Alves[4,5], Sonia Prado-López[4,5,6], Débora Chantada[7], Niko Beerenwinkel[2,3], David Posada[4,5,8], and Ewa Szczurek[1,*]

[1] *Faculty of Mathematics, Informatics and Mechanics, University of Warsaw, Warsaw, Poland*

[2] *Department of Biosystems Science and Engineering, ETH Zurich, 4058 Basel, Switzerland*

[3] *SIB Swiss Institute of Bioinformatics, 4058 Basel, Switzerland*

[4] *CINBIO, Universidade de Vigo, 36310 Vigo, Spain*

[5] *Galicia Sur Health Research Institute (IIS Galicia Sur), SERGAS-UVIGO*

[6] *Institute of Solid State Electronics E362, Technische Universität Wien, Austria*

[7] *Department of Pathology, Hospital Álvaro Cunqueiro, Vigo, Spain*

[8] *Department of Biochemistry, Genetics, and Immunology, Universidade de Vigo, 36310 Vigo, Spain*

[*] *Correspondence: szczurek@mimuw.edu.pl*

Contents

Fig. S1 4

Fig. S2 5

Fig. S3 6

Fig. S4 7

Fig. S5 8

Fig. S6 9

Fig. S7 10

Fig. S8 11

Fig. S9 12

Fig. S10 13

Fig. S11 14

Fig. S12 15

Fig. S13 16

Fig. S14 17

Fig. S15 18

Fig. S16 19

Fig. S17 20

Fig. S18 21

Fig. S19 22

Fig. S20 23

Fig. S21 **24**

Table S1 **25**

Table S2 **26**

Table S3 **27**

Table S4 **28**

Supplementary Note **29**

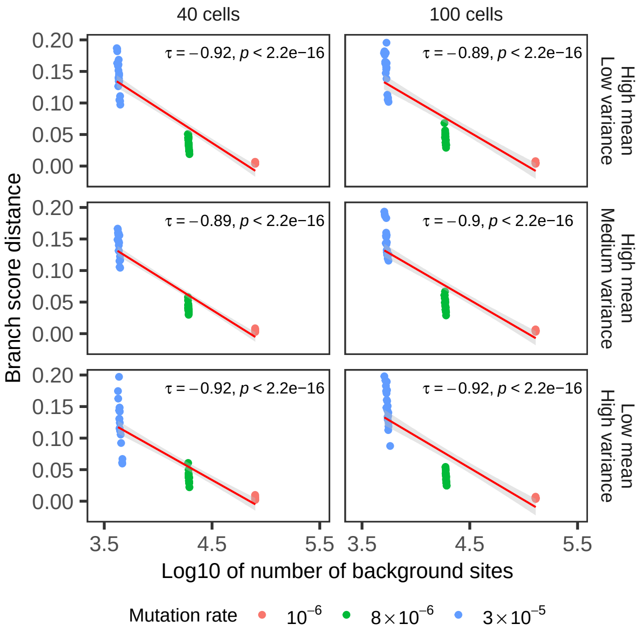

Fig. S1: Correlation plot of the BS distance against the number of background sites in log10 scale. Varying are the number of cells and the coverage quality. BS distance data points are coloured by the corresponding mutation rates. Kendall is the method for computing the correlation coefficient τ, which is invariant to the log transformation of the number of background sites. We choose 0.01 as the significance threshold.

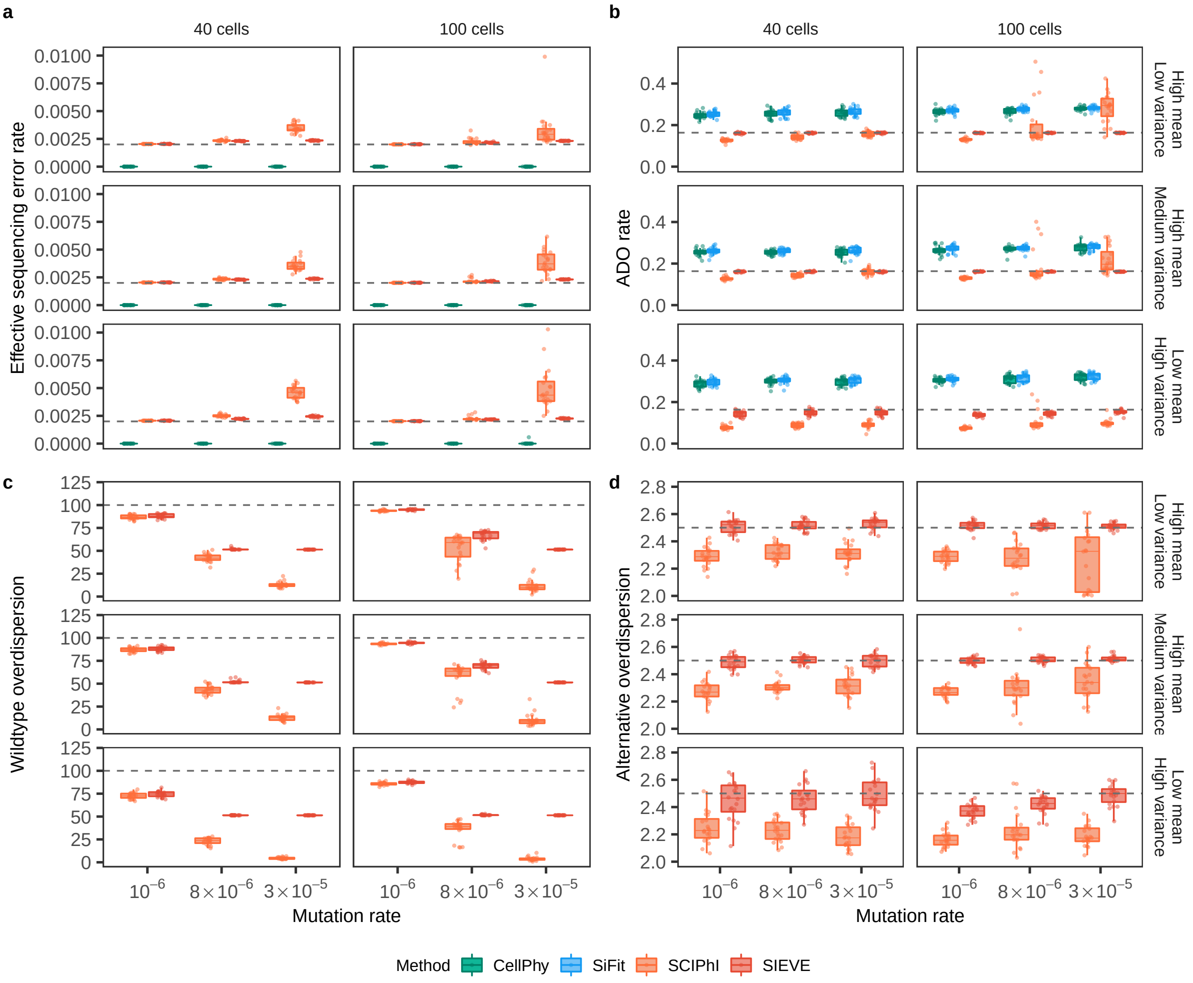

Fig. S2: Additional benchmarking results of the SIEVE model regarding parameter estimates. Each simulation is repeated $n = 20$ times with each repetition denoted by coloured dots. The grey dashed lines represent the ground truth used to generate the simulated data. **a-d**, Box plots of parameter estimation accuracy for four important parameters in the model of raw read counts (Methods): effective sequencing error rate (**a**), ADO rate (**b**), wildtype overdispersion (**c**) and alternative overdispersion (**d**).

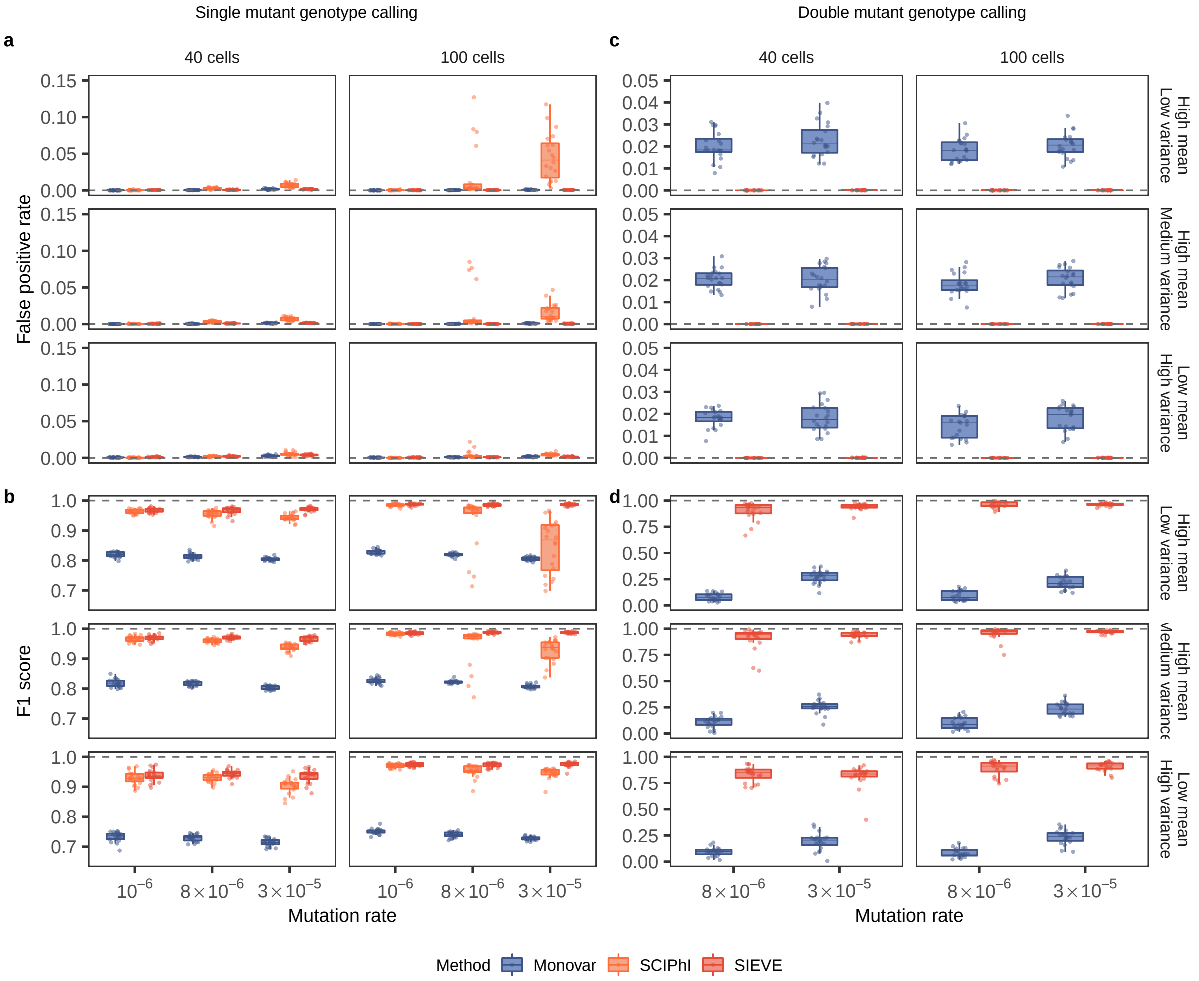

Fig. S3: Additional benchmarking results of the SIEVE model regarding variant calling. Each simulation is repeated $n = 20$ times with each repetition denoted by coloured dots. The grey dashed lines represent the optimal values of each metric. **a-b**, Box plots of the single mutant genotype calling results measured further by the fraction of false positives in the ground truth negatives, i.e., the sum of false positives and true negatives, (false positive rate, **a**) and the harmonic mean of recall and precision (F1 score, **b**). **c-d**, Box plots of the double mutant genotype calling results measured further by false positive rate (**c**) and F1 score (**d**), where the variant calling results when mutation rate is 10^{-6} are omitted as very few double mutant genotypes are generated (less than 0.1%).

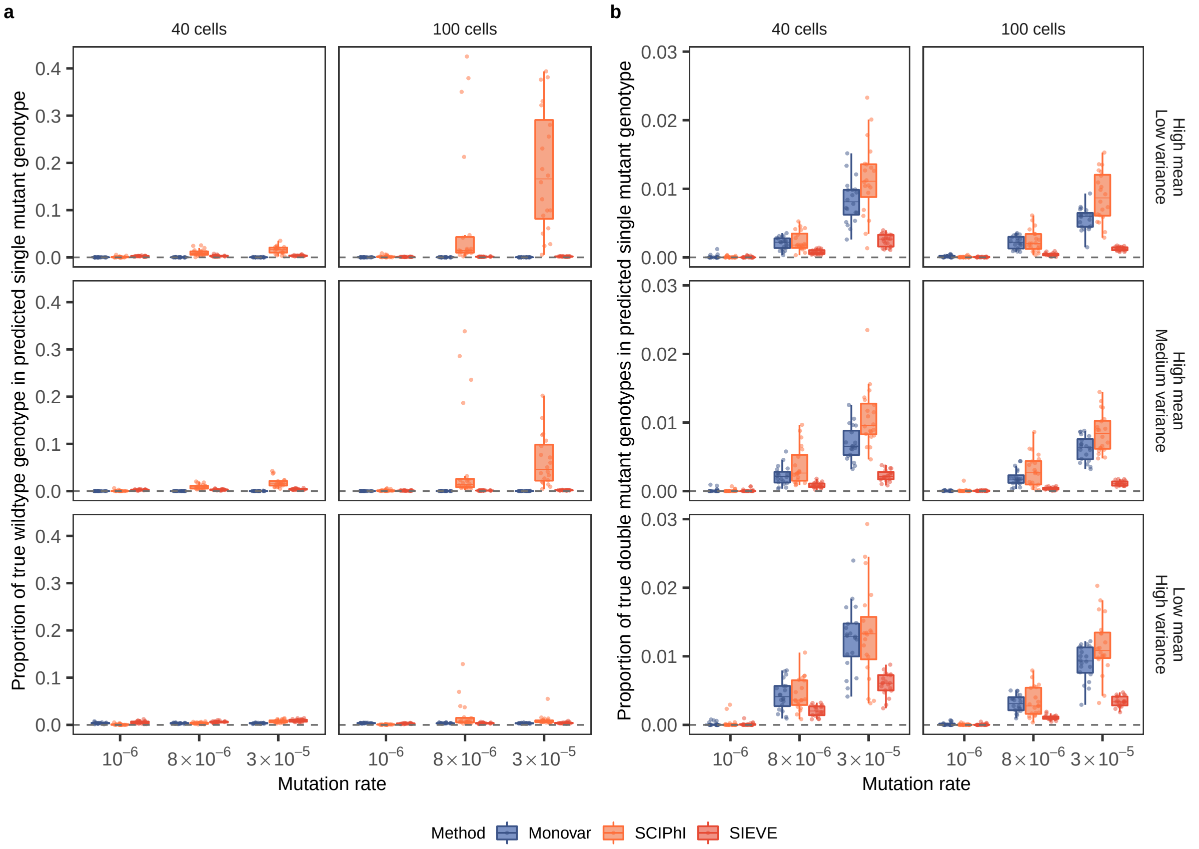

Fig. S4: Types of false positives in single mutant genotype calling. The grey dashed lines represent the optimal proportions of each type. **a-b**, Box plots of the types of false positives in single mutant genotype calling, including the proportion of true wildtype (**a**) and true double mutant genotype (**b**). For single mutant genotype calling, the sum of the precision, the proportion of true wildtype and the proportion of true double mutant genotype is 1.

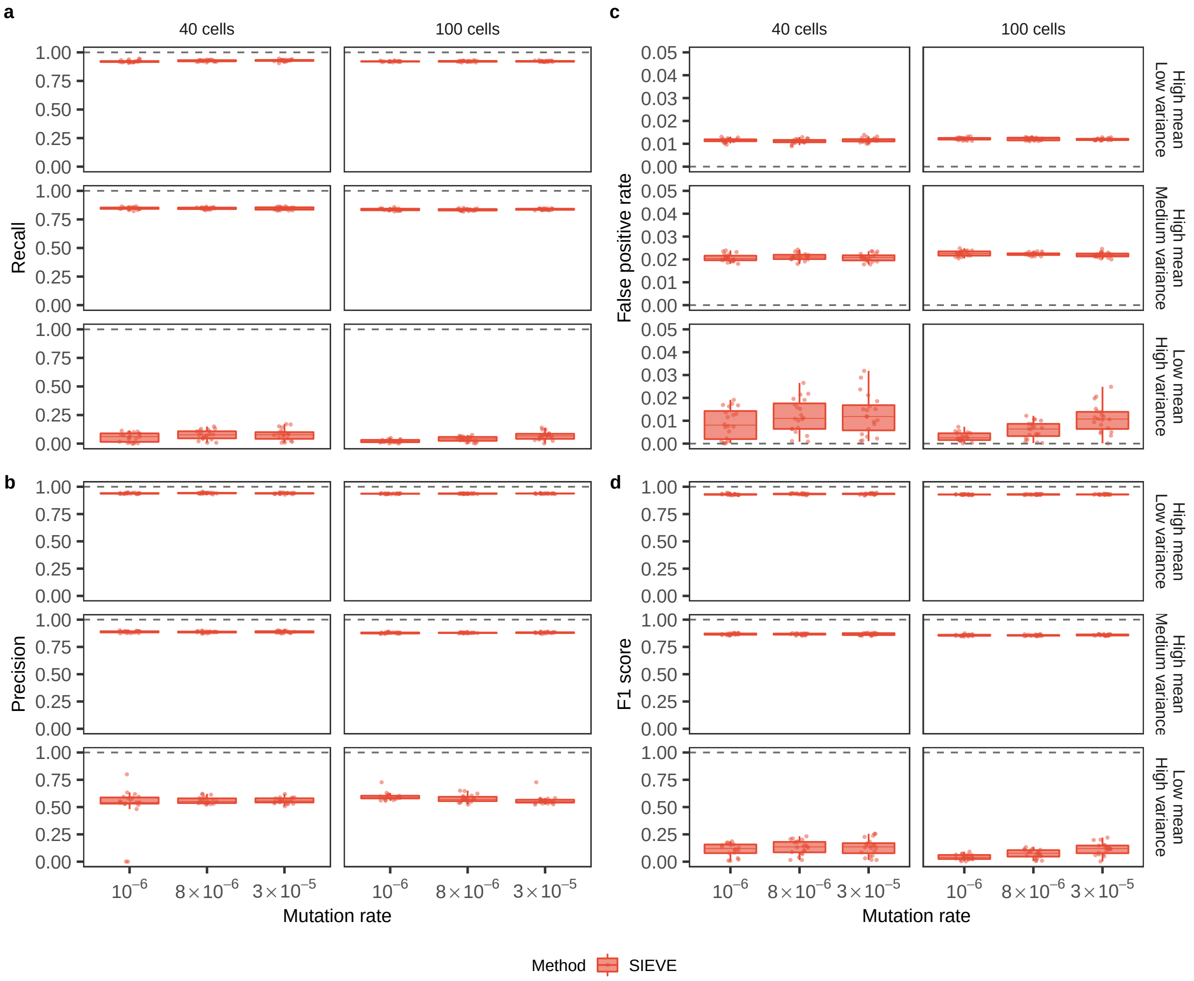

Fig. S5: Benchmarking results of the SIEVE model regarding ADO calling. Each simulation is repeated $n = 20$ times with each repetition denoted by coloured dots. The grey dashed lines represent the optimal values of each metric. **a-d**, Box plots of the ADO calling results measured in recall (**a**), precision (**b**), false positive rate (**c**) and F1 score (**d**).

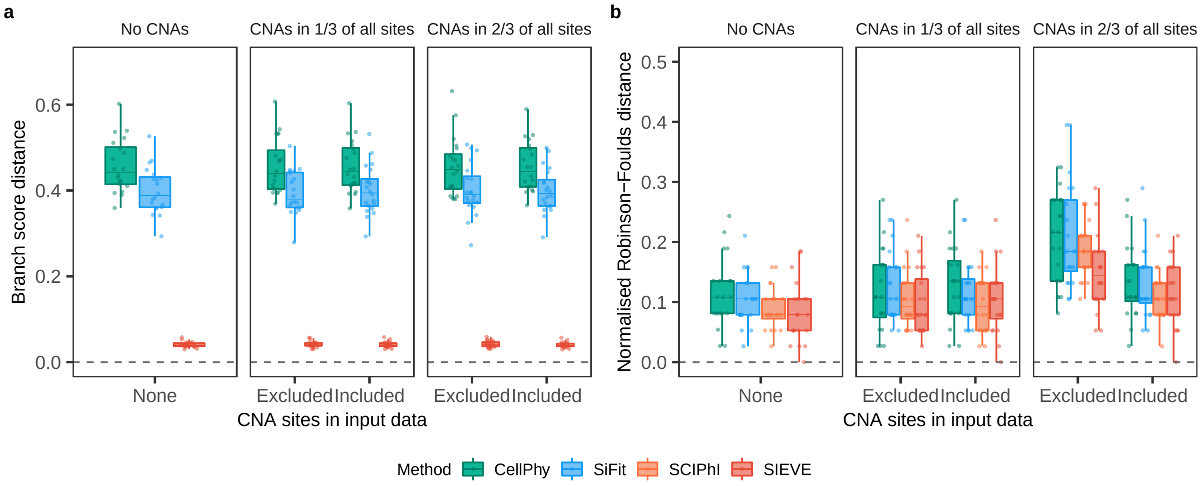

Fig. S6: Tree distance benchmarking results of the SIEVE model considering CNAs. Varying are the prevalence of CNAs in all genomic sites and whether these CNA sites are included or not in the input data. Each simulation is repeated $n = 20$ times with each repetition denoted by coloured dots. The grey dashed lines represent the optimal values of each metric. Box plots comprise medians, boxes covering the interquartile range (IQR), and whiskers extending to 1.5 times the IQR below and above the box. **a-b**, Box plots of the tree inference accuracy measured by the BS distance where the branch lengths are taken into account (**a**) and the normalised RF distance where only tree topology is considered (**b**).

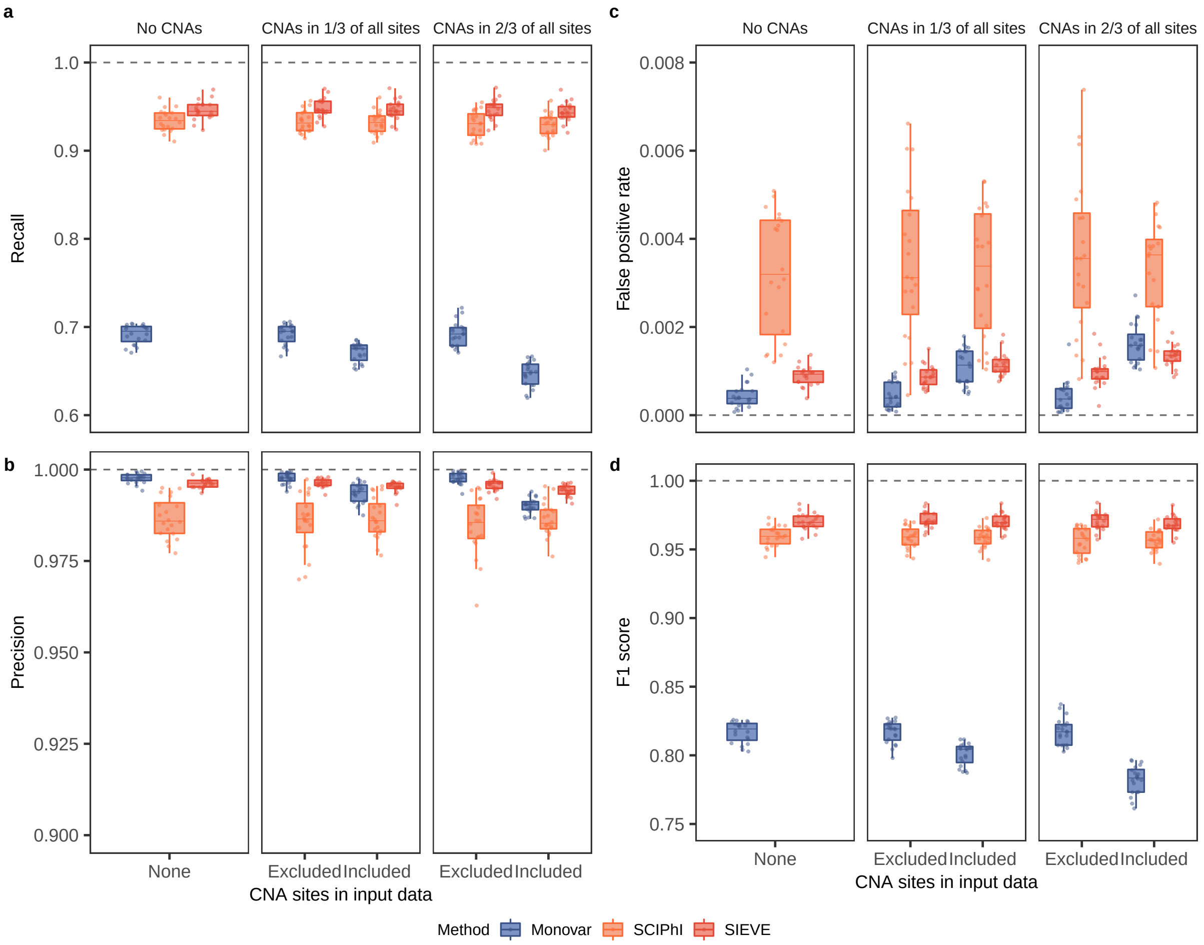

Fig. S7: Single mutant genotype calling results of the SIEVE model considering CNAs. Varying are the prevalence of CNAs in all genomic sites and whether these CNA sites are included or not in the input data. Each simulation is repeated $n = 20$ times with each repetition denoted by coloured dots. The grey dashed lines represent the optimal values of each metric. Box plots comprise medians, boxes covering the interquartile range (IQR), and whiskers extending to 1.5 times the IQR below and above the box. **a-d**, Box plots of the single mutant genotype calling results measured by recall (**a**), precision (**b**), false positive rate (**c**) and F1 score (**d**).

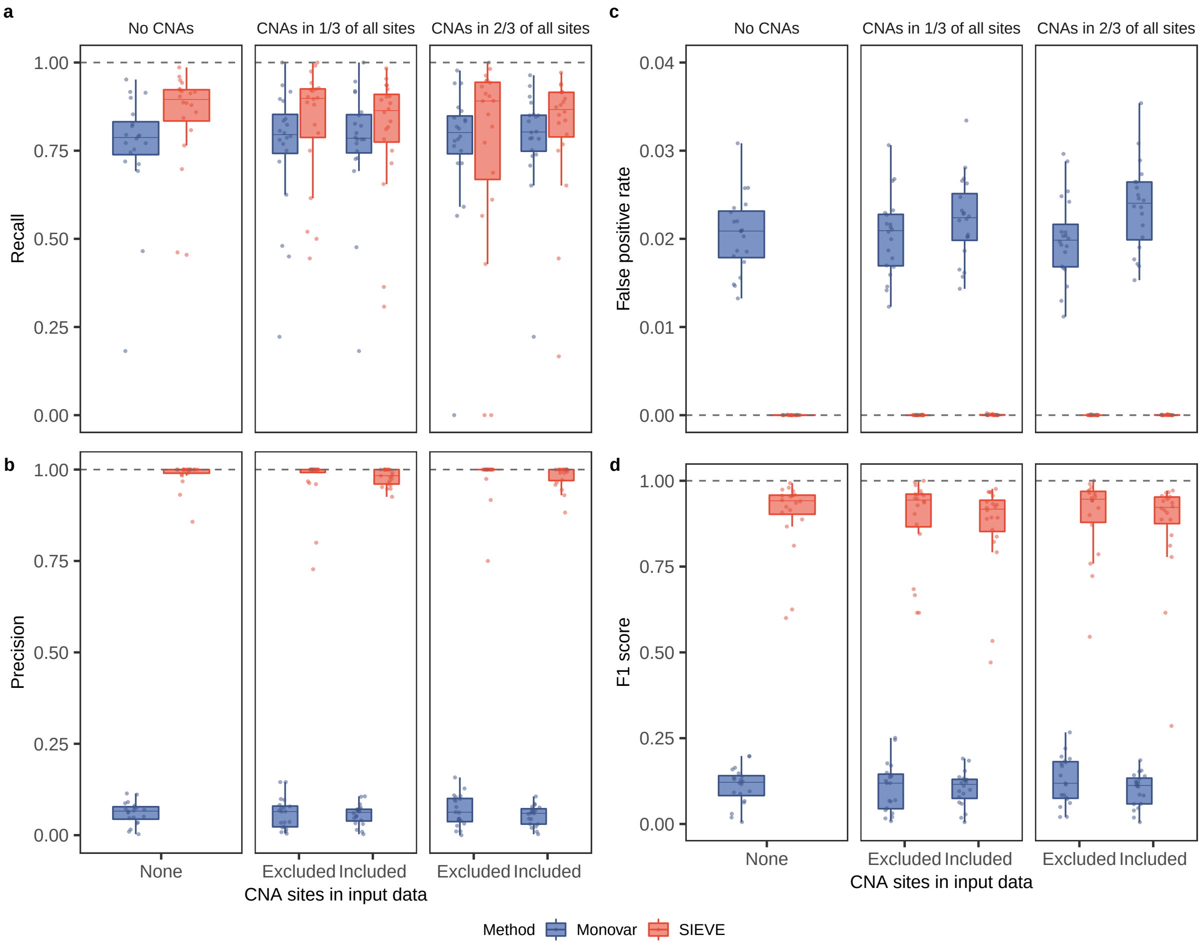

Fig. S8: Double mutant genotype calling results of the SIEVE model considering CNAs. Varying are the prevalence of CNAs in all genomic sites and whether these CNA sites are included or not in the input data. Each simulation is repeated $n = 20$ times with each repetition denoted by coloured dots. The grey dashed lines represent the optimal values of each metric. Box plots comprise medians, boxes covering the interquartile range (IQR), and whiskers extending to 1.5 times the IQR below and above the box. **a-d**, Box plots of the double mutant genotype calling results measured by recall (**a**), precision (**b**), false positive rate (**c**) and F1 score (**d**).

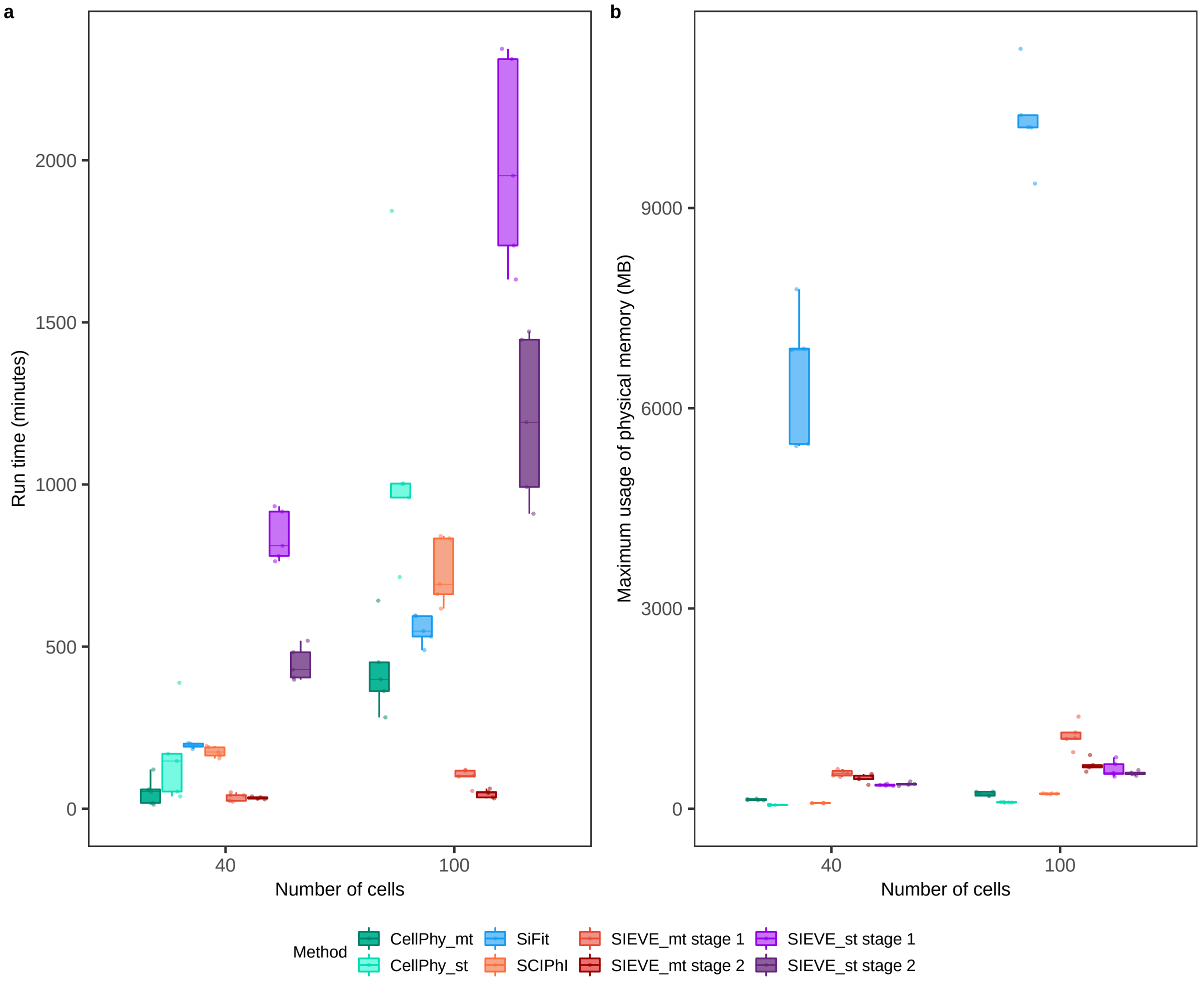

Fig. S9: Run time and memory usage evaluation. Varying is the number of cells. Each simulation is repeated $n = 5$ times with each repetition denoted by coloured dots. SiFit and SCIPhI were run under single-thread mode, while CellPhy and the two stages of SIEVE were run under both single- (CellPhy_st, SIEVE_st stage 1 and SIEVE_st stage 2) and multi-thread (CellPhy_mt and SIEVE_mt stage 1 and SIEVE_mt stage 2) mode. **a-b**, Box plots of efficiency benchmarking results of SIEVE with respect to run time in minutes (**a**) and maximum usage of physical memory in MB (**b**).

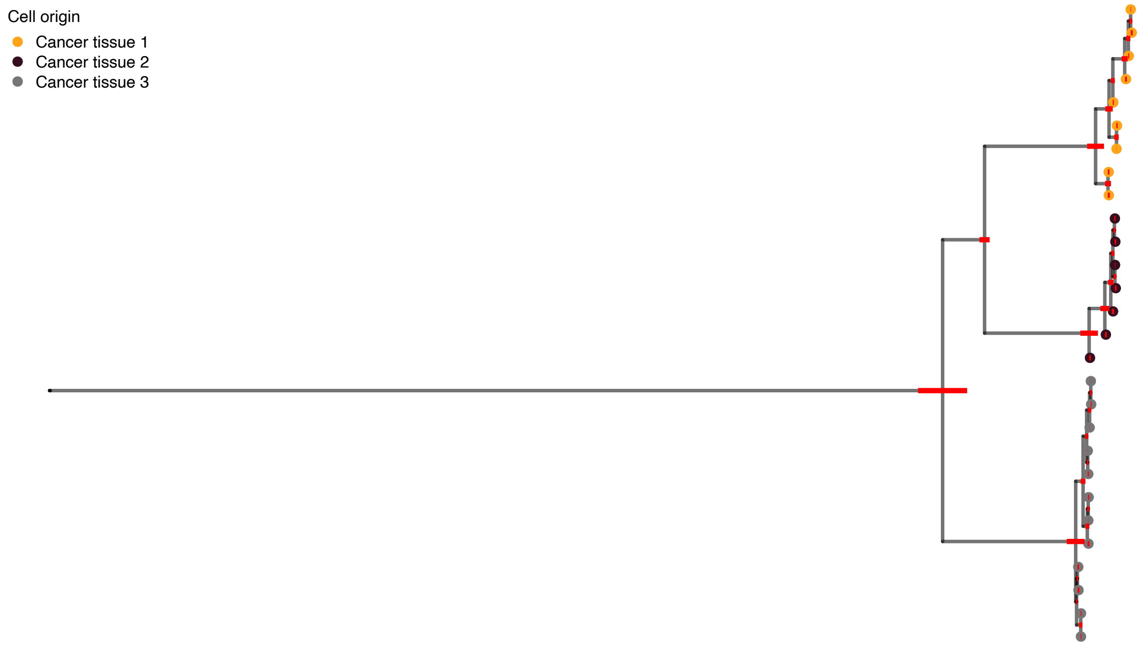

Fig. S10: Illustration of branch lengths of the phylogenetic tree inferred from CRC28 by SIEVE. Shown is exactly the same tree as in Fig. 3, except that cell names, subclone posterior probabilities and gene annotations are removed and no branches are folded. Red bars annotated to internal nodes except the root are the 95% highest posterior density (HPD) intervals of the corresponding branch lengths.

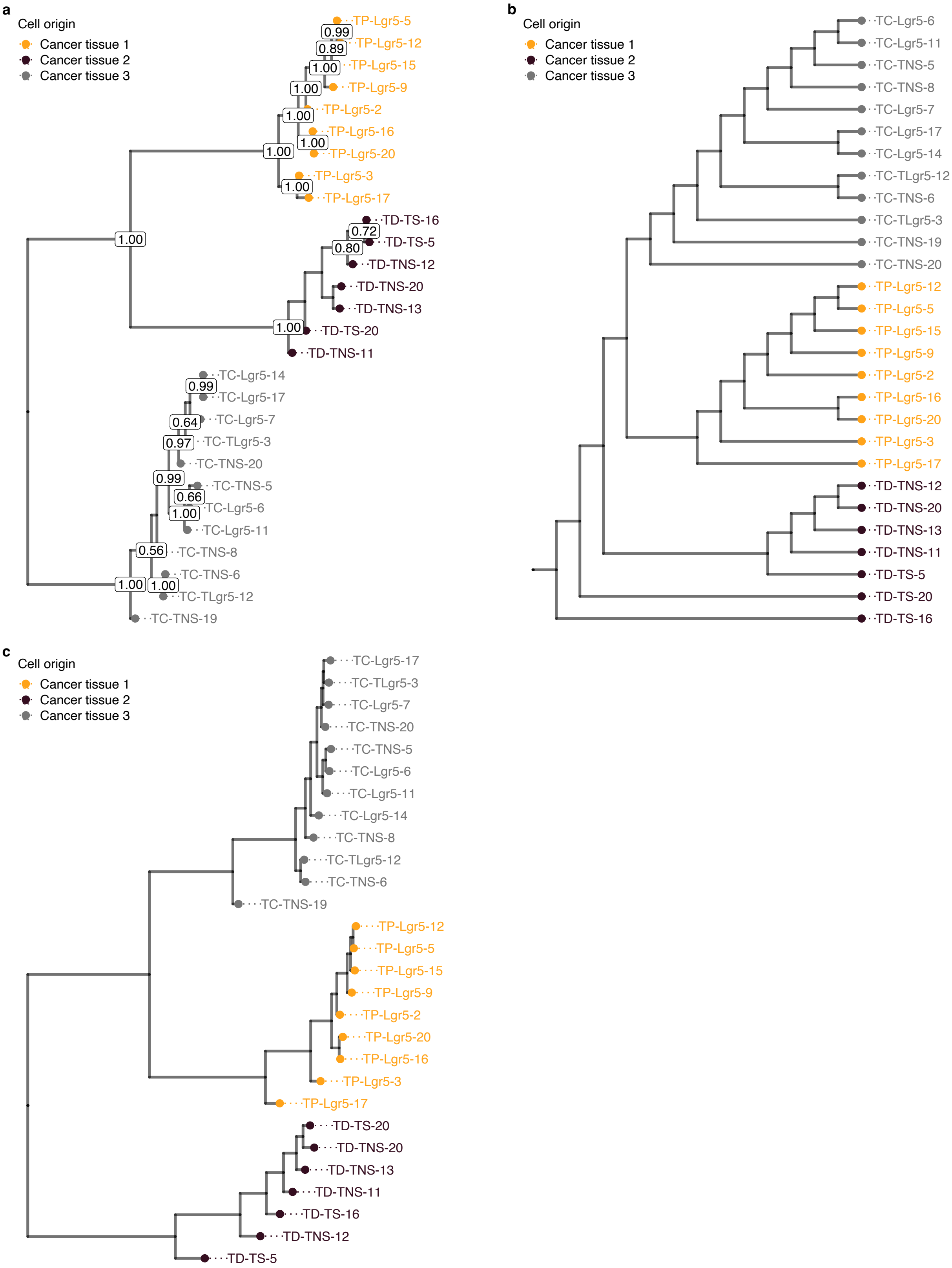

Fig. S11: Illustration of cell phylogenies inferred from CRC28 by other methods. **a-c**, Trees inferred by CellPhy (**a**), SCIPhI (**b**) and SiFit (**c**). CellPhy was run with bootstrap applied, thereby making node supports available. SCIPhI reported only tree topology, not branch lengths.

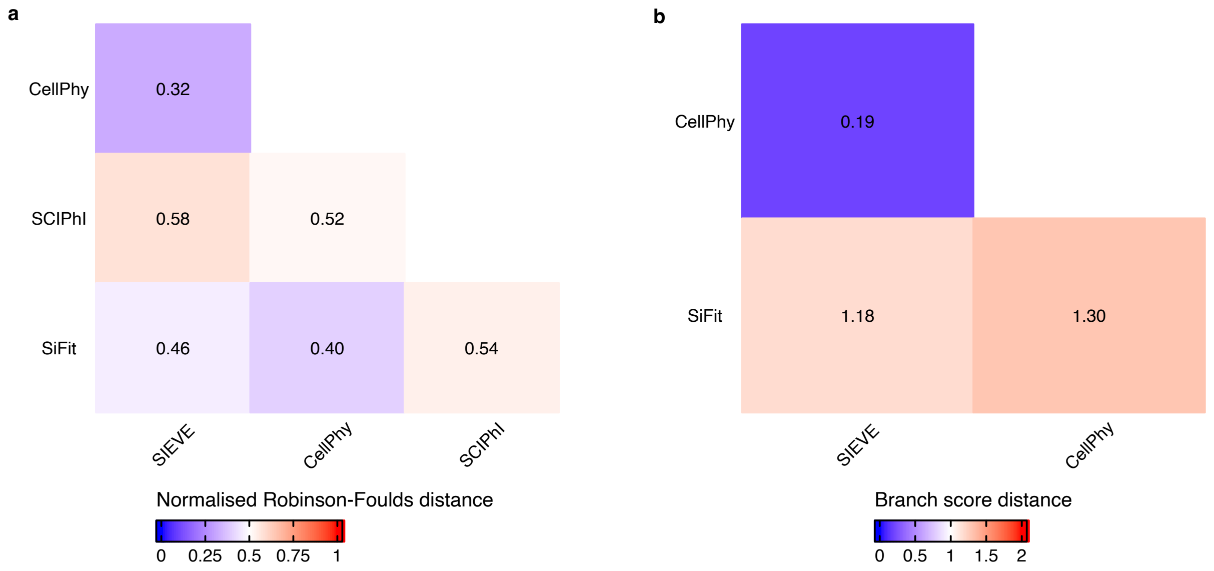

Fig. S12: Heatmaps for pairwise distances of phylogenetic trees inferred from CRC28 by all methods. a-b, Tree distances measured by normalised RF distance (**a**) and BS distance (**b**).

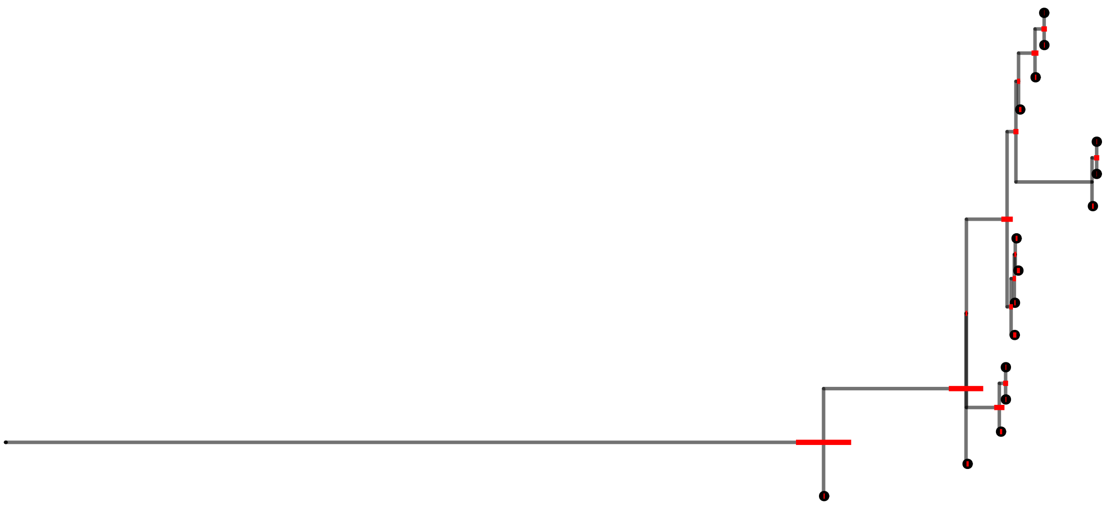

Fig. S13: Illustration of branch lengths of the phylogenetic tree inferred from TNBC16 [42] by SIEVE. Shown is exactly the same tree as in Fig. 4, except that cell names, subclone posterior probabilities and gene annotations are removed and no branches are folded. Red bars annotated to internal nodes except the root are the 95% HPD intervals of the corresponding branch lengths.

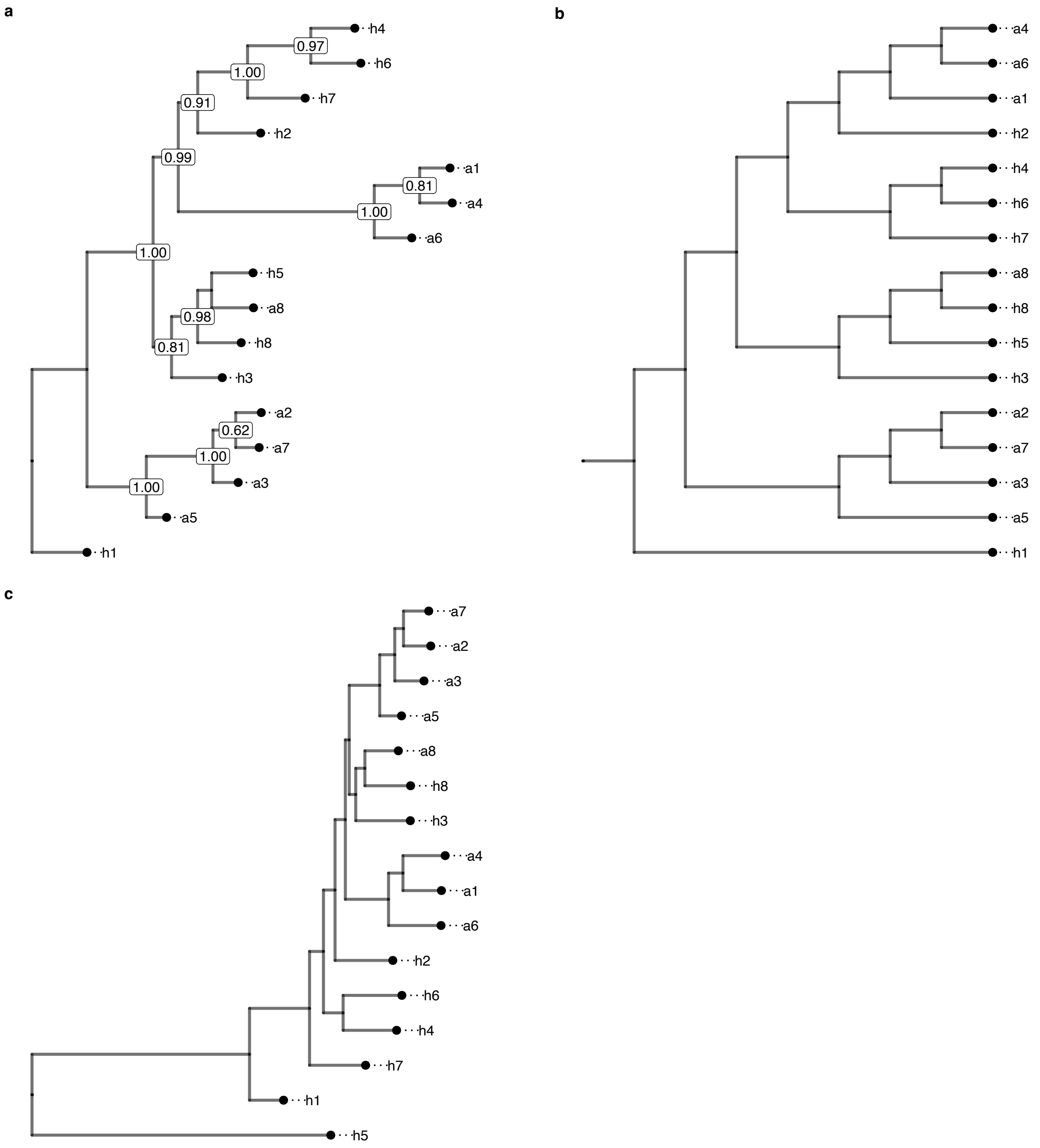

Fig. S14: Illustration of cell phylogenies inferred from TNBC16 [42] by other methods. a-c, Trees inferred by CellPhy (**a**), SCIPhI (**b**) and SiFit (**c**). CellPhy was run with bootstrap applied, thereby making node supports available. SCIPhI reported only tree topology, not branch lengths.

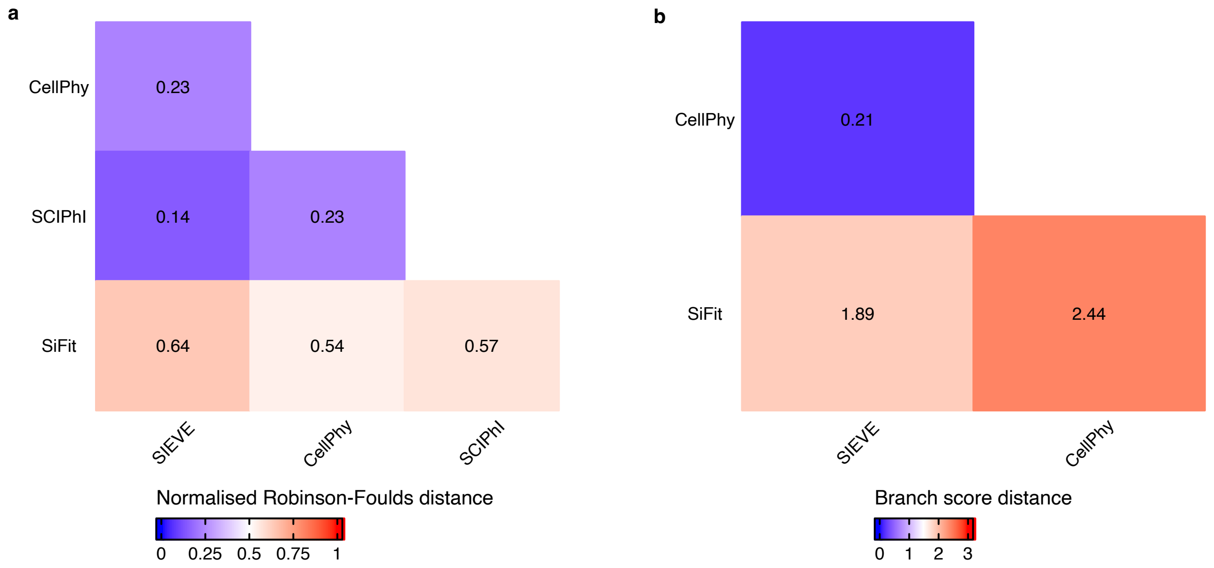

Fig. S15: Heatmaps for pairwise distances of phylogenetic trees inferred from TNBC16 [42] by all methods. a-b, Tree distances measured by normalised RF distance (**a**) and BS distance (**b**).

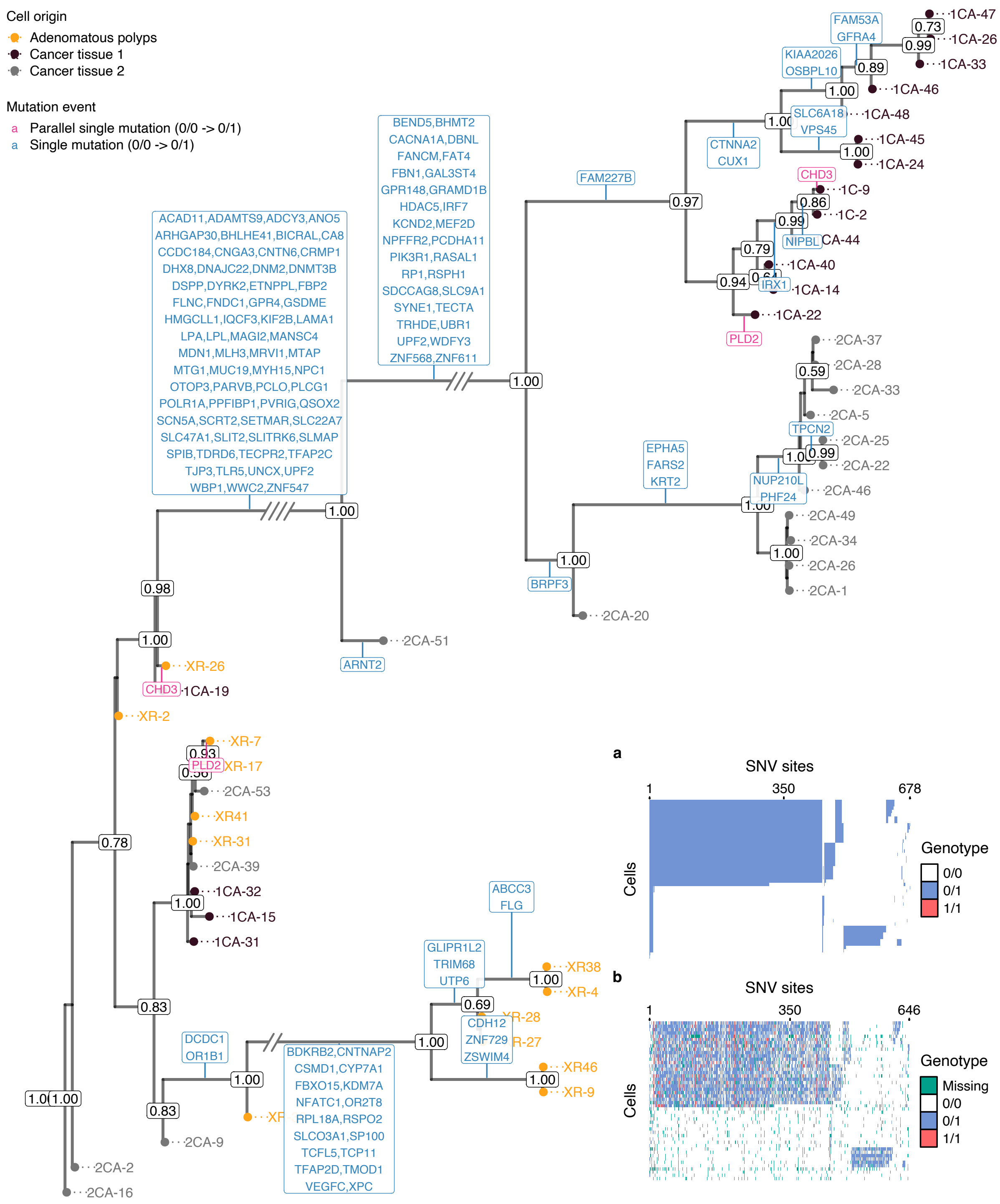

Fig. S16: Results of phylogenetic inference and variant calling for CRC48 [43] dataset. Shown is SIEVE's maximum clade credibility tree. Three exceptionally long branches are folded with the number of slashes proportional to the branch lengths. Cell names are annotated to the leaves of the tree, coloured by the corresponding biopsies. The numbers at each node represent the posterior probabilities (threshold $p > 0.5$). At each branch, non-synonymous mutations are depicted in different colours including single mutations in blue and parallel single mutations in pink. **a-b**, Variant calling heatmap for SIEVE (**a**) and Monovar (**b**). Listed in the legend are the categories of predicted genotypes by each method. Cells in the row are in the same order as that of leaves in the phylogenetic tree.

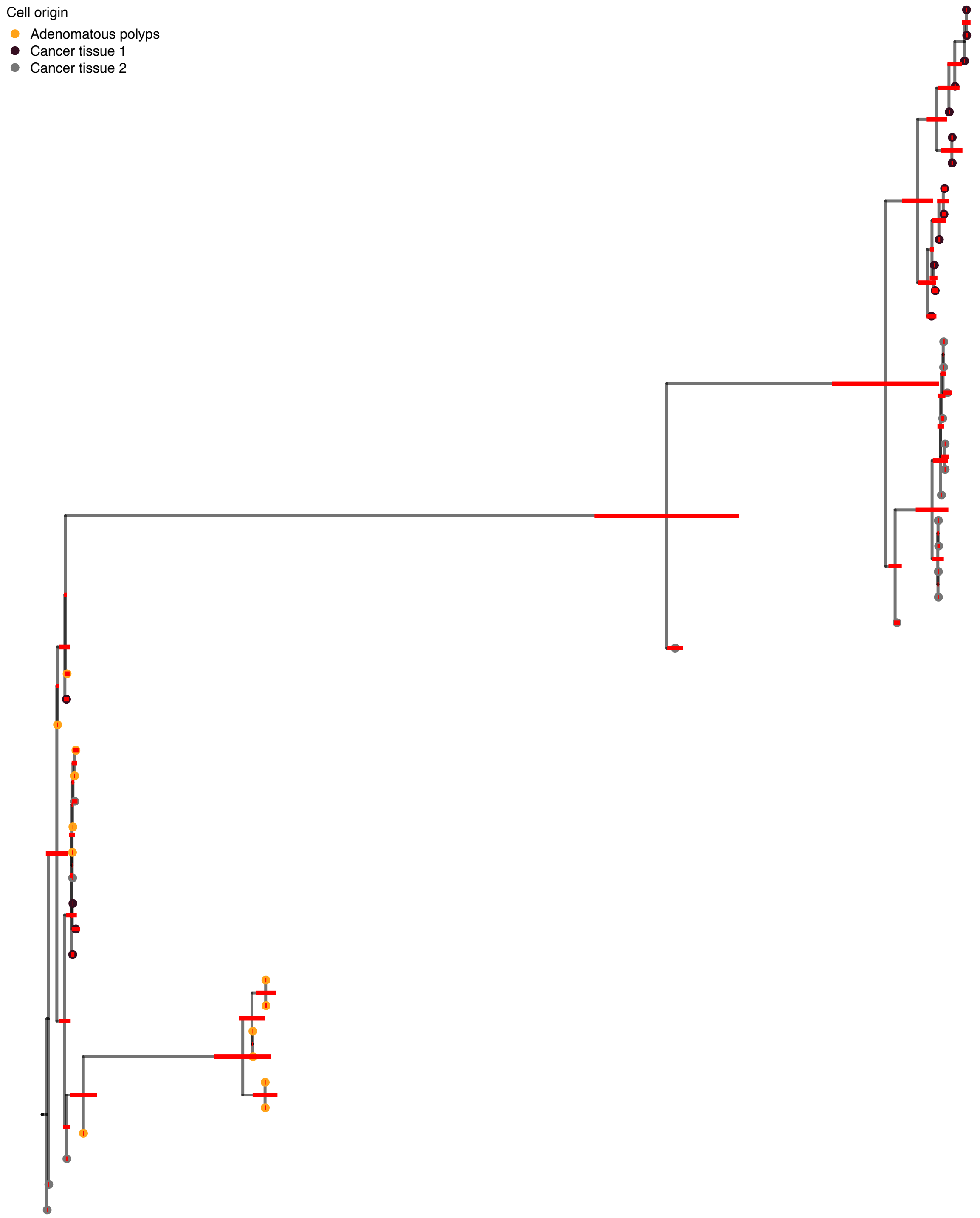

Fig. S17: Illustration of branch lengths of the phylogenetic tree inferred from CRC48 [43] by SIEVE. Shown is exactly the same tree as in Fig. S S16, except that cell names, subclone posterior probabilities and gene annotations are removed and no branches are folded. Red bars annotated to internal nodes except the root are the 95% HPD intervals of the corresponding branch lengths.

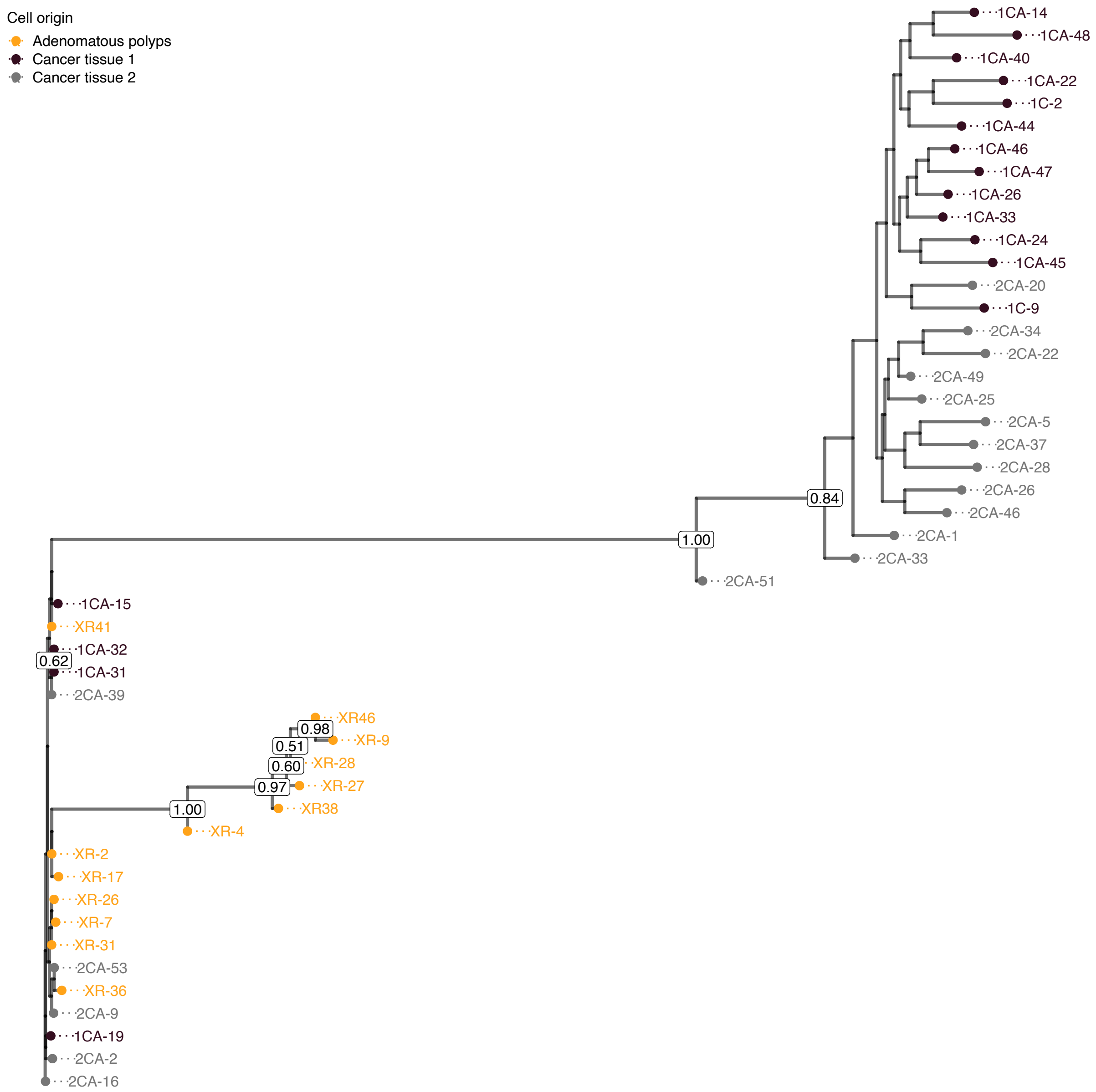

Fig. S18: Illustration of the cell phylogeny inferred from CRC48 [43] by CellPhy. CellPhy was run with bootstrap applied, thereby making node supports available.

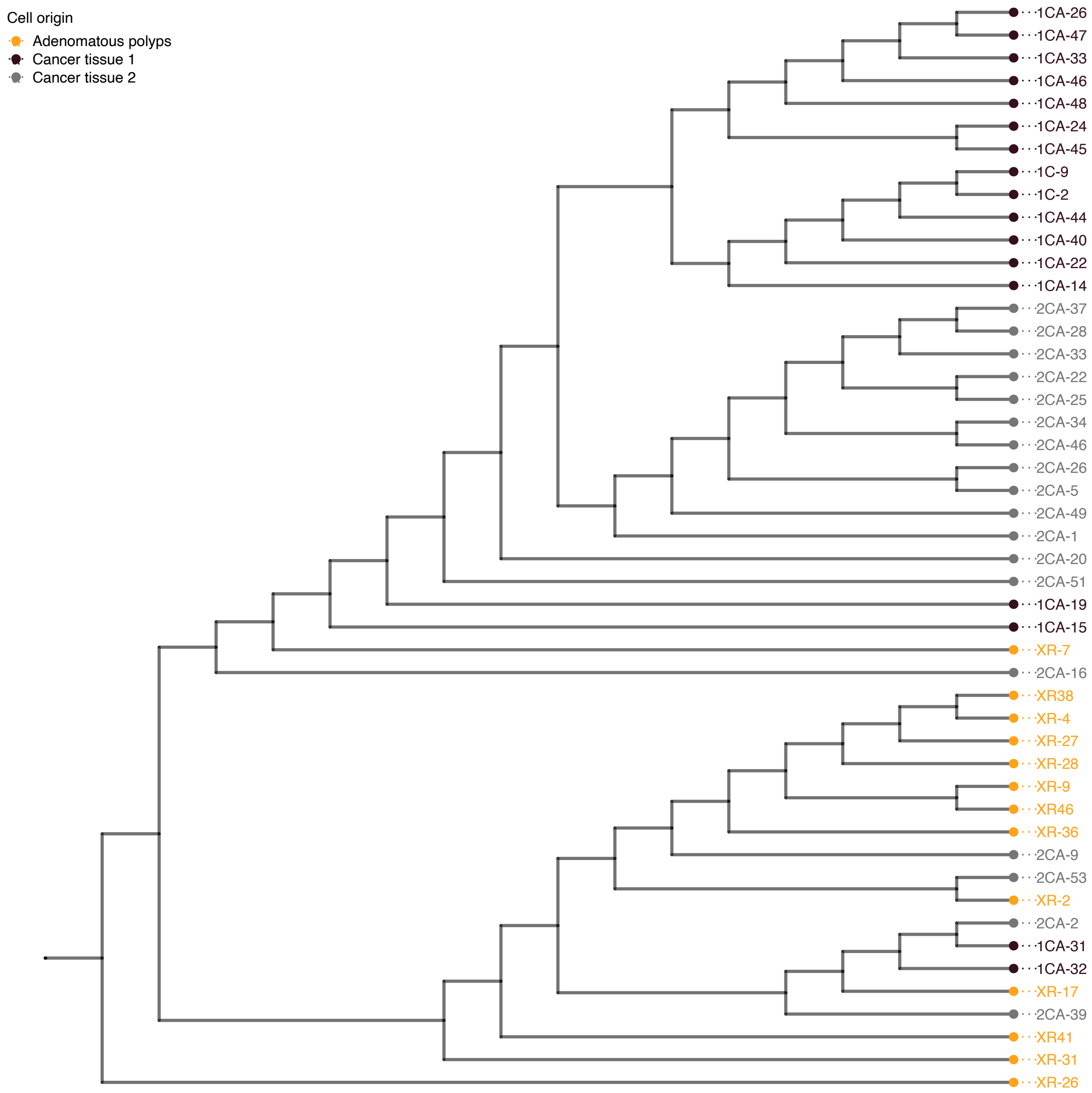

Fig. S19: Illustration of the cell phylogeny inferred from CRC48 [43] by SCIPhI. SCIPhI reported only tree topology, not branch lengths.

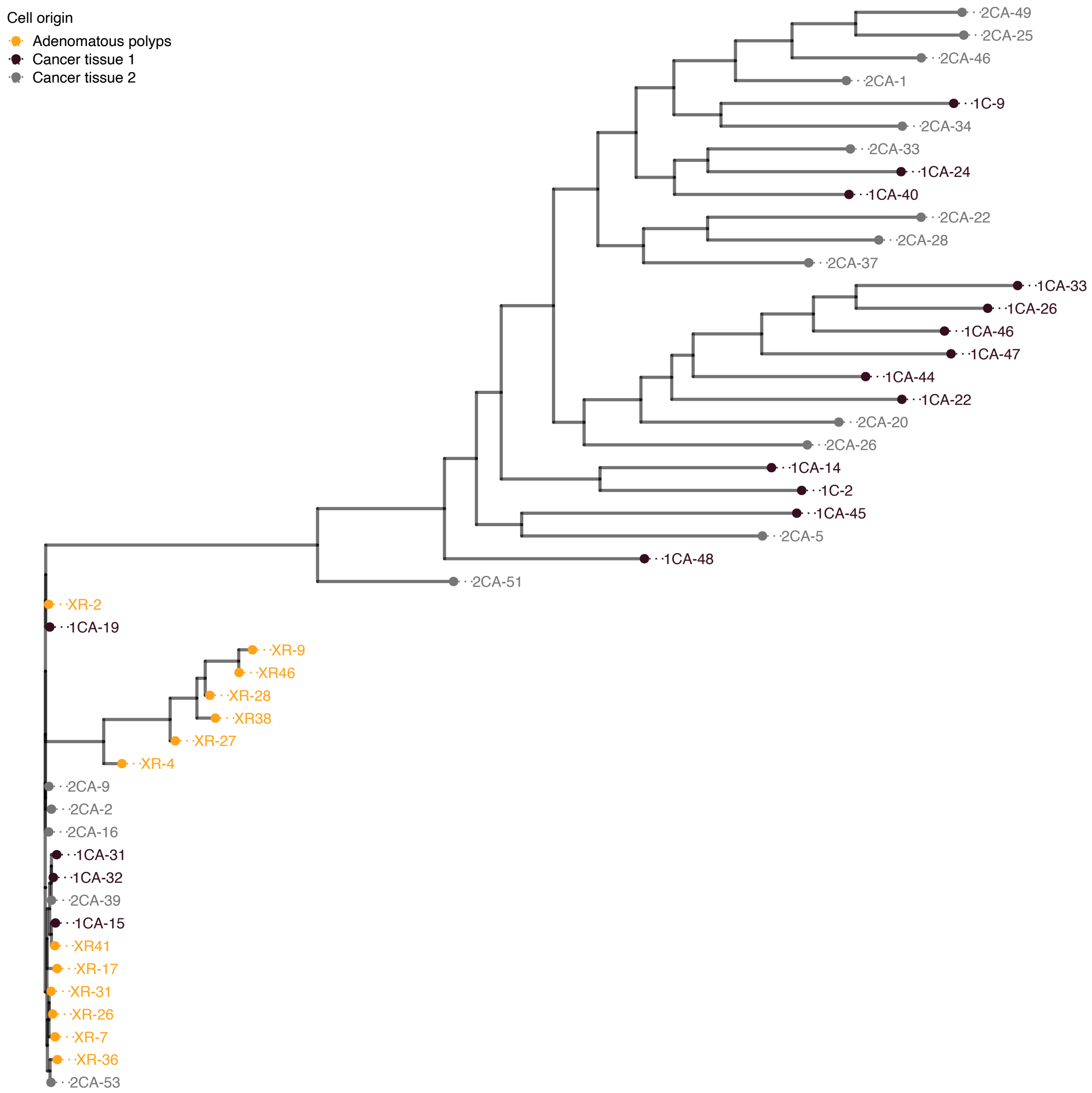

Fig. S20: Illustration of the cell phylogeny inferred from CRC48 [43] by SiFit.

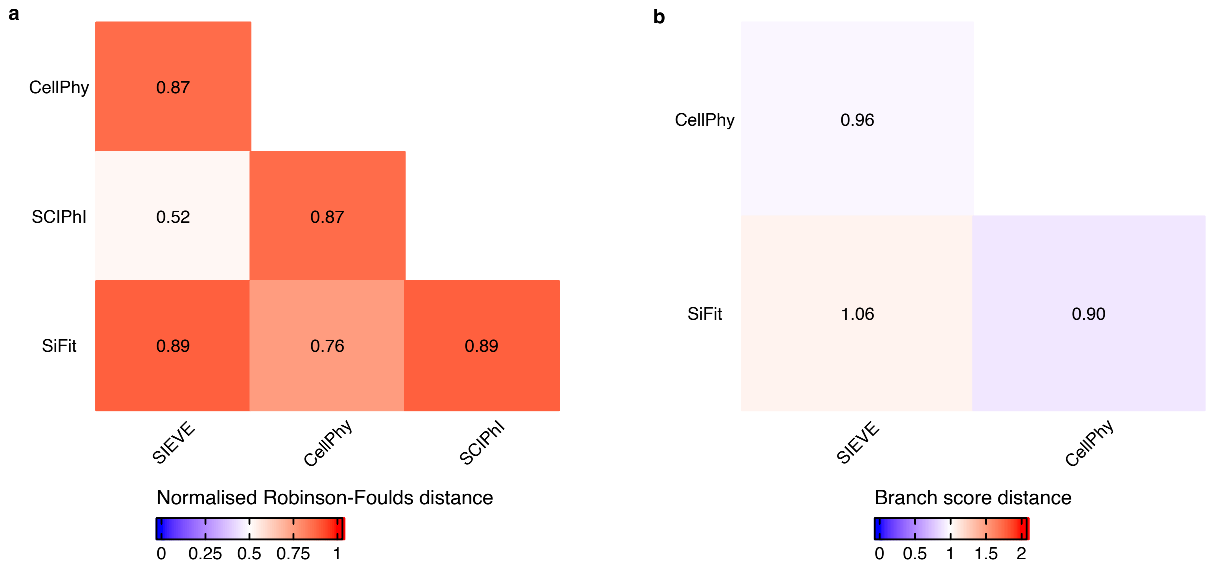

Fig. S21: Heatmaps for pairwise distances of phylogenetic trees inferred from CRC48 [43] by all methods. a-b, Tree distances measured by normalised RF distance (**a**) and BS distance (**b**).

Table S1: Inferred mean and variance of allelic coverage for real datasets.

	Mean of allelic coverage t	Variance of allelic coverage v
CRC28	4.3	19.6
TNBC16	10.2	207.9
CRC48	19.4	635.6

Table S2: The number of (candidate) variant sites and the corresponding number of threads used for CellPhy and SIEVE in the run-time benchmarking. Different number of threads was chosen to achieve the highest efficiency for each method. In particular, when CellPhy was given as many threads as provided to SIEVE, its run time increased.

	No. of cells	No. of (candidate) variant sites	No. of threads
CellPhy	40	774 - 975	4 - 5
	100	932 - 1422	5 - 7
SIEVE	40	786 - 987	31 - 39
	100	961 - 1482	38 - 52

Table S3: Summary of fractions of predicted genotypes by SIEVE and Monovar for three analysed real datasets. Entries marked with NA denote that the corresponding method does not call the specific genotype.

		Missing	0/0	0/1	1/1	$1/1'$
CRC28	SIEVE	NA	25.02%	74.64%	0.28%	0.06%
	Monovar	10.40%	38.09%	46.30%	5.21%	NA
TNBC16	SIEVE	NA	15.54%	75.11%	9.30%	0.05%
	Monovar	10.63%	32.92%	41.58%	14.87%	NA
CRC48	SIEVE	NA	59.48%	40.50%	0.02%	0
	Monovar	4.53%	69.41%	24.13%	1.93%	NA

Table S4: Evolutionary rate matrix used in the simulator to generate the simulated data. Genotypes are encoded with nucleotides rather than numbers.

	A/A	A/C	A/G	A/T	C/C	C/G	C/T	G/G	G/T	T/T
A/A	-1	$1/3$	$1/3$	$1/3$	0	0	0	0	0	0
A/C	$1/6$	-1	$1/6$	$1/6$	$1/6$	$1/6$	$1/6$	0	0	0
A/G	$1/6$	$1/6$	-1	$1/6$	0	$1/6$	0	$1/6$	$1/6$	0
A/T	$1/6$	$1/6$	$1/6$	-1	0	0	$1/6$	0	$1/6$	$1/6$
C/C	0	$1/3$	0	0	-1	$1/3$	$1/3$	0	0	0
C/G	0	$1/6$	$1/6$	0	$1/6$	-1	$1/6$	$1/6$	$1/6$	0
C/T	0	$1/6$	0	$1/6$	$1/6$	$1/6$	-1	0	$1/6$	$1/6$
G/G	0	0	$1/3$	0	0	$1/3$	0	-1	$1/3$	0
G/T	0	0	$1/6$	$1/6$	0	$1/6$	$1/6$	$1/6$	-1	$1/6$
T/T	0	0	0	$1/3$	0	0	$1/3$	0	$1/3$	-1

Supplementary Note

Commands for data preprocessing

Similar preprocessing procedure was done for all real datasets. TNBC16 dataset is shown as an example below.

File containing raw sequencing data information:

```
### acc-cell.txt (A text file containing cell sra ids for download and \
corresponding cell names)


SRR1163012      a1
SRR1163013      a2
SRR1163019      a3
SRR1163026      a4
SRR1163027      a5
SRR1163034      a6
SRR1163035      a7
SRR1163043      a8
SRR1163053      h1
SRR1163070      h2
SRR1163074      h3
SRR1163083      h4
SRR1163084      h5
SRR1163091      h6
SRR1163095      h7
SRR1163148      h8
SRR1163149      TNBC_n1
SRR1163150      TNBC_n2
SRR1163151      TNBC_n3
SRR1163152      TNBC_n4
SRR1163153      TNBC_n5
SRR1163154      TNBC_n6
SRR1163155      TNBC_n7
```

```
SRR1163156      TNBC_n8
SRR1163157      TNBC_n9
SRR1163158      TNBC_n10
SRR1163159      TNBC_n11
SRR1163160      TNBC_n12
SRR1163161      TNBC_n13
SRR1163162      TNBC_n14
SRR1163163      TNBC_n15
SRR1163164      TNBC_n16
SRR1163508      TNBC_Pop_Normal
SRR1298936      TNBC_Pop_Tumor
```

Other files or constants needed for preprocessing:

```
REF=hs37d5.fa
DBSNP=dbsnp_138.b37.vcf
INDELS=Mills_and_1000G_gold_standard.indels.b37.vcf
INDELS2=1000G_phase1.indels.b37.vcf
### $SLURM_ARRAY_TASK_ID - chromosomes.
SLURM_ARRAY_TASK_ID = 1-22
```

Downloading Data according to SRA using SRA-Toolkit.

```
module load sra-toolkit/2.9.2-centos_linux64
cat acc-cell.txt | while read -r sra_id cellname;do
fastq-dump --split-files $sra_id
done
```

Fastq Quality Control using CutAdapt.

```
module load gcccore/6.4.0 cutadapt/1.18-python-3.7.0
cat acc-cell.txt | while read -r sra_id cellname;do
cutadapt --minimum-length 70 \
        -a AGATCGGAAGAGCACACGTCTGAACTCCAGTCACNNNNNNATCTCGTATGCCGTCTTCTGCTTG  \
        -o Processing/${sra_id}.trimmed_1.fastq.gz \
```

```
        Raw_Data/${sra_id}_1.fastq.gz > ${sra_id}_Cutadapt.txt
done
```

Alignment using BWA.

```
module load gcccore/6.4.0 cutadapt/1.18-python-3.7.0
module load gcc/6.4.0 bwa/0.7.17
module load picard/2.18.14

cat acc-cell.txt | while read -r sra_id cellname;do

ID=${cellname}
SM=$(echo ${cellname} | cut -d "_" -f1)
PL=$(echo "ILLUMINA")
LB=$(echo "KAPA")
PU=‘zcat Raw_Data/${cellname}_1.fastq.gz | head -n1 | \
        sed ’s/[:].*//’ | sed ’s/@//’ | sed ’s/ /_/’‘
echo "SAMPLE: "${cellname}" ID: "${ID}" SM: "${SM}
RG="@RG\\tID:${ID}\\tSM:${SM}\\tPL:${PL}\\tLB:${LB}\\tPU:${PU}"

bwa mem -t 10 \
        -R ${RG} \
        $REF \
        ./Processing/${sra_id}.trimmed_1.fastq.gz \
        > Processing/${cellname}.sam

java -Xmx18g -jar $EBROOTPICARD/picard.jar SortSam \
        I=Processing/${cellname}.sam \
        TMP_DIR=Processing/ \
        O=Processing/${cellname}.sorted.bam \
        CREATE_INDEX=true \
        SORT_ORDER=coordinate
done
```

Mark Duplicates using Picard Tools.

```
module load picard/2.18.14

cat acc-cell.txt | while read -r sra_id cellname;do

java -Xmx35g -jar $EBROOTPICARD/picard.jar MarkDuplicates \

        I=Processing/$cellname.sorted.bam \

        TMP_DIR=Processing/ \

        O=Processing/${cellname}.dedup.bam \

        CREATE_INDEX=true \

        VALIDATION_STRINGENCY=LENIENT \

        M=Processing/Duplicates_${cellname}.txt

done
```

Realignment using GATK.

```
module load gatk/3.7-0-gcfedb67


sample_bams=$(ls Processing/*.dedup.bam)

bams_in=$(echo $sample_bams | sed 's/ / -I /g')

echo $sample_bams |sed 's/ /\n/g' | sed 's/.dedup.bam//g' | \

        awk -v chr=$SLURM_ARRAY_TASK_ID \

        '{print $0".dedup.bam\t"$0".real."chr".bam"}' | \

        sed 's/Processing\///' > W32.$SLURM_ARRAY_TASK_ID.map


java -Djava.io.tmpdir=Processing/ -Xmx25G \

        -jar $EBROOTGATK/GenomeAnalysisTK.jar \

        -T RealignerTargetCreator \

        -I $bams_in \

        -o W32.$SLURM_ARRAY_TASK_ID.intervals \

        -R $REF \

        -known $INDELS \

        -known $INDELS2 \

        -L $SLURM_ARRAY_TASK_ID
```

```
java -Djava.io.tmpdir=Processing/ -Xmx25G \
        -jar $EBROOTGATK/GenomeAnalysisTK.jar \
        -T IndelRealigner \
        -known $INDELS \
        -known $INDELS2 \
        -I $bams_in \
        -R $REF \
        -targetIntervals W32.$SLURM_ARRAY_TASK_ID.intervals \
        -L $SLURM_ARRAY_TASK_ID \
        --nWayOut W32.$SLURM_ARRAY_TASK_ID.map \
        --maxReadsForRealignment 1000000
```

Recalibration using GATK.

```
module load gatk/4.0.10.0
cat acc-cell.txt | while read -r sra_id cellname;do
gatk --java-options "-Xmx24G -Djava.io.tmpdir=Processing/" BaseRecalibrator \
        -I Processing/$cellname.real.$SLURM_ARRAY_TASK_ID.bam \
        -O Processing/$cellname.recal.$SLURM_ARRAY_TASK_ID.table \
        -R $REF \
        --known-sites $DBSNP \
        --known-sites $INDELS


gatk --java-options "-Xmx24G -Djava.io.tmpdir=Processing/" ApplyBQSR \
        -R $REF \
        -I Processing/$cellname.real.$SLURM_ARRAY_TASK_ID.bam \
        --bqsr Processing/$cellname.recal.$SLURM_ARRAY_TASK_ID.table \
        -O Processing/$cellname.recal.$SLURM_ARRAY_TASK_ID.bam
done
```

Bam to mpileup using samtools.

```
module load samtools/1.9
ls ../Processing/*.recal.$SLURM_ARRAY_TASK_ID.bam | \
```

```
        grep -v "Tumor" > bampath.$SLURM_ARRAY_TASK_ID.txt
samtools mpileup --no-BAQ --min-BQ 13 --max-depth 10000 --min-MQ 40 \
        -r $SLURM_ARRAY_TASK_ID -b bampath.$SLURM_ARRAY_TASK_ID.txt \
        -f $REF -o W32.$SLURM_ARRAY_TASK_ID.mpileup
```
