# Supplementary material for: SIEVE: joint inference of single-nucleotide variants and cell phylogeny from single-cell DNA sequencing data
Source: Genome Biol. 2022 Nov 30;23:248. doi: 10.1186/s13059-022-02813-9 (PMC9714196; doi:10.1186/s13059-022-02813-9)
Supplement: Supplementary file 2 — Additional file 2. Review history. [file 13059_2022_2813_MOESM2_ESM.docx]

Review History

**First round of review**

**Reviewer 1**

**Were you able to assess all statistics in the manuscript, including the appropriateness of statistical tests used?**

Yes: The statistical model appeared appropriate, although I ask in the comments to the author for clarification on one point regarding identifying candidate SNVs.

**Were you able to directly test the methods?**

No

**Comments to author:**

This paper presents a new method, SIEVE, for joint single nucleotide variant (SNV) calling and phylogeny inference for single-cell sequence data. Variant calling and cell phylogenetics from single cell sequence data are both problems getting a lot of attention right now in the computational biology literature right now. They have become crowded areas for tool-building, but they are not solved problems and ever-improving sequencing technologies and growing interest in cell lineage tracing have created a need for better methods and new ideas. The main innovations of the present work are to combine variant calling and phylogeny construction into a single analysis and to employ an improved model of phylogeny branch length inference. These are plausible arguments for both innovations as ways to improve the state of the art in the domain. It also improves on at least some currently popular software in using a finite sites model. And it offers some nice practical utility through integration into the BEAST framework.

The mathematical models behind the work are well explained and appear appropriate to the problem and technically sound. Algorithmically, the method also appears appropriate for the problem, with some defensible approximations and a suitable Markov chain Monte Carlo strategy for model fitting.

The paper validates the methods with comparisons to a variety of competing tools, using both simulated data and two real test cases. The authors consider a reasonable set of alternative methods and I believe the design of data sets and the performance measures considered are fair. The results show very good results in comparison to alternatives, and are nicely analyzed. The analysis of real data also appears well done, showing the methods to be practical for real data, to yield at least plausible results, and to provide some novel insights into the biology of the test cases examined.

Overall, SIEVE appears to be a sound and valuable contribution to the field that is shown to improve significantly on the prior art. I believe it should be of interest primarily to methods developers in this space but also to potential users of somatic cell lineage analysis tools, such as those conducting cancer genome studies. Nonetheless, a few points raise questions for me:

1. I did not understand why the method requires a set of pre-determined candidate SNV sites as input, as I would have thought that finding those sites is part of the job of a variant caller. The authors mention a separate program that does this candidate determination, but details are sparse on how it works and why this is not done internal to the main SIEVE variant caller. It would seem more logical for the method to take something like SAM format data as input and identify the SNV sites itself. Could the authors elaborate on why it is done this why and precisely what the model and algorithm are used for the variant candidate inference? Is this design just for efficiency or would something else go wrong if one input every sequenced site as a candidate SNV site?

2. The model is focused only on SNVs, which is a defensible choice for a tool in this space, but even if one is only using SNVs as phylogenetic markers, it is still questionable to ignore copy number variation. The model appears to assume all DNA examined is diploid, which is often going to be an incorrect assumption in tumor genomes. That may be a fine approximation, but I think it at least needs to be considered and some analysis done of how the method would perform in the presence of copy number variation. If, for example, one ran the method on tetraploid data, would it produce reasonable results? Or does one need to preprocess to exclude aneuploid tumors or non-diploid regions to use the methods?

3. I was intrigued that the authors mentioned using the BEAST2 relaxed molecular clock in the real data analysis and would appreciate some elaboration on this point. One of the potentially important limitations of the main model described in the paper would seem to be the assumption of a fixed mutation rate. This assumption is problematic for cancers, which commonly acquire mutations that change the subsequent mutation rate. If I am reading correctly that implementing this software through BEAST2 gives an easy way to relax that assumption, then I think that specifically may be another significant advantage of this software over alternatives that is worth highlighting. If so, I think it is worth explaining why this model was used on the real data and exploring a bit further whether it showed anything interesting. For example, where you see lineages of seemingly different ages, can any of that be connected to mutations in DNA mismatch repair genes, POLD/E, etc.?

4. I think in general the paper could use more comment on the model assumptions and especially where the work makes assumptions that we would expect to violated in practice. For example, the mutation model would seem to assume mutations are not under selection, when at least some cancer mutations can be assumed to be under strong selection. Likewise, the model assumes mutations are independent, when there are many reasons one might see dependence between mutations in cancers (localized mutation bursts like kataegis, mutually exclusive mutations, elevated mutability of regions of high copy number, etc.). Would any such issues be expected to create problems of data interpretation. I would suspect these exceptions to apply to small enough fraction of mutations that they will not affect results much, but if so it is still worth considering model assumptions and pointing out that they can be ignored.

5. On a more minor note, page 26, the parameters xi_1, xi_2, psi_1, and psi_2 seem oddly specific without a clear reason why those particular values are used. Could you elaborate on how those were derived?

6. I noted just one minor typographical error: On p. 23, line 22, in the phrase "J cells and K genotype in G", genotype should be plural.

**Reviewer 2**

**Were you able to assess all statistics in the manuscript, including the appropriateness of statistical tests used?**

Yes: The methods are clearly reported in this paper

**Were you able to directly test the methods?**

Yes

**Comments to author:**

- Are the methods appropriate to the aims of the study, are they well described, and are necessary controls included?

The methods are appropriately described since this is a methodological paper and the focus is SIEVE, the probabilistic graphical model presented by the authors.

- Are the conclusions adequately supported by the data shown?

The paper presents SIEVE that, according to simulations carried out from an independent data-generation tool, it seems to work reasonably well.

- Are sufficient details provided to allow replication and comparison with related analyses that may have been performed?

SIEVE is made available even though I could not attempt at reproducing the results of the authors.

- Is the method likely to be of broad utility? Is any software component easy to install and use? Please indicate briefly the novel features and/or advantages of the method, and/or please reference the relevant publications and which methods, if any, it should be compared with.

The focus of SIEVE is to support, within BEAST2, the computation of a full Bayesian posterior on the phylogeny of a sample while accounting for the Finite State Assumption. The proposed method is elegantly defined and the sampling MCMC algorithm seems clear; the integration with BEAST2 facilitates the usability of the tool which benefits from a set of standardised algorithms (e.g., Felsenstein's pruning algorithm) already available in BEAST2. SIEVE is heavily focused for cancer researchers trying to infer advanced phylogenies from their data, and I think SIEVE is a direct competitor of CellPhy, a rival method leveraging maximum-likelihood phylogeny reconstruction and RAxML-NG. So, RAxML-NG and BEAST2 have now these two cancer-specific phylogeny reconstruction methods available, with the advantage of SIEVE also calling mutations which CellPhy does not.

Regarding comparisons, I think the method has been compared to the relevant tools in the field, especially CellPhy - I would suggest the authors to report computational times of the comparison, keeping in mind that they should include bootstrap time computations from CellPhy as correct analogous of full Bayesian posterior estimates. One thing I did not get very much clearly (maybe in real data, or on specific simulated examples), what are the major tree-structure differences we can expect to find employing a prior in SIEVE, that we have against an ML approach - is there any substantial difference in the type of evolutionary story we tell, or in the end is more a statistical difference?

The paper should also be largely improved on the actual real-data application part. The main criticism is that this FSA assumption seems to be very important for the TNBC example, and the authors say that

"By mapping non-synonymous mutations to the internal branches, we identified different types of mutation events (Methods), including several violations of the ISA, such as back mutations and parallel mutations."

I do not know if I missed that (I did not find it in the Methods), but I think that a clear account of which events are associated to back-mutations and parallel evolution are important, as well as a potential explanation of why that would happen. Moreover, I would like to see how much a tree with ISA would differ from the one with FSA, and in general I would like to see the trees from different methods to be compared rigoroously. For instance, in one case it is claimed that because the posterior over SIEVE's tree is higher than one on the tree returned by another method, then the SIEVE tree is better. THat is true assuming the bayesian priors in SIEVE make sense -- which I can agree with -- but a more thorough presentation of this difference is important to understand how much actual "evolutionary biology" we miss by using the wrong method.

Right now - if I am not mistaken - the most sophisticate biological digression by the authors is that cells form clades based on anatomical collection in the CRC case studies. Is there anything else we can discover from this complex phylogenomic methods and their advanced statistical properties? I think this is a key aspect that would make the paper more appealing to the GB readership.

- Is the paper of broad interest to others in the field, or of outstanding interest to a broad audience of biologists?

I think the paper is of interest to a subset of the cancer community, namely only to the people carrying out sc-WGS or similar assays to apply this method.

**Authors Response**

**Point-by-point responses to the reviewers’ comments:**

**Reviewer #1:** This paper presents a new method, SIEVE, for joint single nucleotide variant (SNV) calling and phylogeny inference for single-cell sequence data. Variant calling and cell phylogenetics from single cell sequence data are both problems getting a lot of attention right now in the computational biology literature right now. They have become crowded areas for tool-building, but they are not solved problems and ever-improving sequencing technologies and growing interest in cell lineage tracing have created a need for better methods and new ideas. The main innovations of the present work are to combine variant calling and phylogeny construction into a single analysis and to employ an improved model of phylogeny branch length inference. These are plausible arguments for both innovations as ways to improve the state of the art in the domain. It also improves on at least some currently popular software in using a finite sites model. And it offers some nice practical utility through integration into the BEAST framework.

The mathematical models behind the work are well explained and appear appropriate to the problem and technically sound. Algorithmically, the method also appears appropriate for the problem, with some defensible approximations and a suitable Markov chain Monte Carlo strategy for model fitting.

The paper validates the methods with comparisons to a variety of competing tools, using both simulated data and two real test cases. The authors consider a reasonable set of alternative methods and I believe the design of data sets and the performance measures considered are fair. The results show very good results in comparison to alternatives, and are nicely analyzed. The analysis of real data also appears well done, showing the methods to be practical for real data, to yield at least plausible results, and to provide some novel insights into the biology of the test cases examined.

Overall, SIEVE appears to be a sound and valuable contribution to the field that is shown to improve significantly on the prior art. I believe it should be of interest primarily to methods developers in this space but also to potential users of somatic cell lineage analysis tools, such as those conducting cancer genome studies. Nonetheless, a few points raise questions for me:

1. I did not understand why the method requires a set of pre-determined candidate SNV sites as input, as I would have thought that finding those sites is part of the job of a variant caller. The authors mention a separate program that does this candidate determination, but details are sparse on how it works and why this is not done internal to the main SIEVE variant caller. It would seem more logical for the method to take something like SAM format data as input and identify the SNV sites itself. Could the authors elaborate on why it is done this why and precisely what the model and algorithm are used for the variant candidate inference? Is this design just for efficiency or would something else go wrong if one input every sequenced site as a candidate SNV site?

*Thank you for this comment. Phylogenetic inference using the full set of all possible sites is computationally infeasible. Thus, the predetermination of the candidate sites is crucial for model performance, as it limits the number of sites where the variation may occur to a number which is usually much smaller compared to the number of all possible sites. Note that SCIPhI, similarly to SIEVE, also uses these sites as candidates and performs variant calling for the pre-selected sites. Other methods, such as CellPhy and SiFit, require more information at input, namely the actual variant calls (specification that which variant sites are mutated in which cells).*

*In short, our candidate variant identification tool (DataFilter) adapts the methodology of candidate sites selection from SCIPhI, making it suitable for the extended functionality of SIEVE. Based on this input and the raw read data, SIEVE performs the actual variant calling.*

*DataFilter and SIEVE are both implemented by ourselves and could be viewed as a joint tool, although implemented in two steps. Indeed, DataFilter takes pileup files as input and outputs the candidate sites, which are later utilized by SIEVE. The only reason why these two steps are implemented separately is purely technical, namely DataFilter is implemented in C++, and SIEVE in Java.*

*This explanation and more details about how DataFilter works and why it is needed was now added to a new Methods subsection “Candidate site identification” in page 20.*

2. The model is focused only on SNVs, which is a defensible choice for a tool in this space, but even if one is only using SNVs as phylogenetic markers, it is still questionable to ignore copy number variation. The model appears to assume all DNA examined is diploid, which is often going to be an incorrect assumption in tumor genomes. That may be a fine approximation, but I think it at least needs to be considered and some analysis done of how the method would perform in the presence of copy number variation. If, for example, one ran the method on tetraploid data, would it produce reasonable results? Or does one need to preprocess to exclude aneuploid tumors or non-diploid regions to use the methods?

*Indeed, the model assumes a diploid genome and this assumption was now listed in a new subsection “Summary of model assumptions”, in the Methods (page 34).*

*To not violate the diploid genome assumption, one should preprocess the data to exclude aneuploid tumors or non-diploid regions. On the other hand, this comes with the cost of removing sites in these regions. Leaving such sites introduces discrepancy with the assumption, but may give more statistical power for model inference. Thus, there is a tradeoff associated with the decision of including or excluding copy number-altered regions, and we leave this decision and the potential data preprocessing step to the user.*

*As suggested by the Reviewer, to guide such decisions, we comprehensively investigated the performance of SIEVE and all compared models (CellPhy, SiFit, and SCIPhI) in the presence of moderate and large copy number aberrations (CNAs) in the simulated data, including deletions, both with and without the sites that were affected by the copy number changes. Overall, the CNAs do decrease the performance of all models, but to only a limited extent. The accuracy of the tree inference worsened when CNAs were present in large amounts and excluded from the data. In terms of variant calling, CNAs did not affect SIEVE or SCIPhi much, but worsened the performance of Monovar. These findings are illustrated in the new Supplementary Fig. 6-8 and described in a new subsection “SIEVE accurately infers cell phylogeny and calls variants with the presence of copy number aberrations (CNAs)” in the Results (page 10).*

3. I was intrigued that the authors mentioned using the BEAST2 relaxed molecular clock in the real data analysis and would appreciate some elaboration on this point. One of the potentially important limitations of the main model described in the paper would seem to be the assumption of a fixed mutation rate. This assumption is problematic for cancers, which commonly acquire mutations that change the subsequent mutation rate. If I am reading correctly that implementing this software through BEAST2 gives an easy way to relax that assumption, then I think that specifically may be another significant advantage of this software over alternatives that is worth highlighting. If so, I think it is worth explaining why this model was used on the real data and exploring a bit further whether it showed anything interesting. For example, where you see lineages of seemingly different ages, can any of that be connected to mutations in DNA mismatch repair genes, POLD/E, etc.?

*Thank you for this comment. For model inference, we are using the functionality of the BEAST 2, which comes either the strict molecular clock or the relaxed clock model. For the real cancer data we used the relaxed clock model since, indeed, it is better suited to the nature of the process of cancer evolution. We now highlight this feature of the model in a new subsection “Summary of evolutionary features accounted for by the model” in the Methods (page 35).*

*As pointed out by the Reviewer, for some of the real datasets, we find imbalances of branch lengths, which may be associated with different mutation rates along the branches. We now discuss this aspect for the tree inferred for CRC48 (Supplementary Fig. 16), which, in contrast to CRC28 (Fig. 3), showed large imbalance of branch lengths. For that tree, we found gene MLH3 in the trunk of the subtree connecting all tumor cells, which could have resulted in the large imbalance of the entire reconstructed tree (Results, page 16).*

4. I think in general the paper could use more comment on the model assumptions and especially where the work makes assumptions that we would expect to violated in practice. For example, the mutation model would seem to assume mutations are not under selection, when at least some cancer mutations can be assumed to be under strong selection. Likewise, the model assumes mutations are independent, when there are many reasons one might see dependence between mutations in cancers (localized mutation bursts like kataegis, mutually exclusive mutations, elevated mutability of regions of high copy number, etc.). Would any such issues be expected to create problems of data interpretation. I would suspect these exceptions to apply to small enough fraction of mutations that they will not affect results much, but if so it is still worth considering model assumptions and pointing out that they can be ignored.

*Thank you for this insightful comment. We now added to the Methods a new subsection “Summary of model assumptions” (page 34). In particular, we discuss the assumption of independent sites, which is made by our and all existing models for phylogenetic inference that we are aware of. This assumption means that a change in each site is independent of changes in other sites.*

*Our model indeed does not account for phenomena such as mutually exclusive mutations or mutational signatures. As correctly pointed out by the Reviewer, sets of mutually exclusive mutations are very small relative to the total number of variant sites and are not expected to affect the performance of our model in any manner. Importantly, the fact that the model does not account for these phenomena does not mean that it makes assumptions that are in conflict with them. For example, mutual exclusivity of two mutations could be observed when one mutation would affect cells in one subbranch of the phylogenetic tree, and another would affect the rest of the tree.*

*Similarly, kataegis and mutation bursts or higher mutability regions are not phenomena that stand in conflict with the independent site assumptions of our model, as we do not assume that all sites have the exact same mutation rate along the genome (similarly to all other phylogenetic methods).*

*We would also like to explain that SIEVE as a mutation model does not assume anything at all about neutrality nor selection. As for mutations, it is only considering the conditional probabilities of change of genotype states to one another.*

5. On a more minor note, page 26, the parameters xi_1, xi_2, psi_1, and psi_2 seem oddly specific without a clear reason why those particular values are used. Could you elaborate on how those were derived?

*We now explain the way these parameters were fixed in page 31 below equation 27. Briefly, the means, namely xi_1 and xi_2, were log-transformed to follow the convention in BEAST 2, and their values before transformation were less oddly specific, namely 150 and 10, respectively. Along with psi_1 and psi_2, these parameters were hand picked so that both the prior distributions covered a large range of possible values for w_1 and w_2, resulting in a largely uninformative prior for these values. This is explained in line 615, page 31.*

6. I noted just one minor typographical error: On p. 23, line 22, in the phrase "J cells and K genotype in G", genotype should be plural.

*Thank you for noticing this. The typo has been corrected.*

**Reviewer #2:** - Are the methods appropriate to the aims of the study, are they well described, and are necessary controls included?

The methods are appropriately described since this is a methodological paper and the focus is SIEVE, the probabilistic graphical model presented by the authors.

- Are the conclusions adequately supported by the data shown?

The paper presents SIEVE that, according to simulations carried out from an independent data-generation tool, it seems to work reasonably well.

- Are sufficient details provided to allow replication and comparison with related analyses that may have been performed?

SIEVE is made available even though I could not attempt at reproducing the results of the authors.

- Is the method likely to be of broad utility? Is any software component easy to install and use? Please indicate briefly the novel features and/or advantages of the method, and/or please reference the relevant publications and which methods, if any, it should be compared with.

The focus of SIEVE is to support, within BEAST2, the computation of a full Bayesian posterior on the phylogeny of a sample while accounting for the Finite State Assumption. The proposed method is elegantly defined and the sampling MCMC algorithm seems clear; the integration with BEAST2 facilitates the usability of the tool which benefits from a set of standardised algorithms (e.g., Felsenstein's pru demening algorithm) already available in BEAST2. SIEVE is heavily focused for cancer researchers trying to infer advanced phylogenies from their data, and I think SIEVE is a direct competitor of CellPhy, a rival method leveraging maximum-likelihood phylogeny reconstruction and RAxML-NG. So, RAxML-NG and BEAST2 have now these two cancer-specific phylogeny reconstruction methods available, with the advantage of SIEVE also calling mutations which CellPhy does not.

Regarding comparisons, I think the method has been compared to the relevant tools in the field, especially CellPhy - I would suggest the authors to report computational times of the comparison, keeping in mind that they should include bootstrap time computations from CellPhy as correct analogous of full Bayesian posterior estimates.

*Thank you for your comment. We have added a new Supplementary Fig. 9, which compares the run times and the memory usage of the competing methods. This figure is described in a new subsection “SIEVE achieves favorable run times and low memory usage in the default, multi-thread mode” in the Results (page 11). We observe that SIEVE in its default, multi-thread mode, outperforms other Bayesian methods. Moreover, it performs comparably well as CellPhy does, which is a maximum likelihood-based method, especially for larger numbers of cells, when bootstrap is applied. SIEVE achieved this performance using more threads than CellPhy (comparison included in a new Supplementary Table 2). However, reserving as many threads for CellPhy as for SIEVE did not increase its performance. Also, we found that SIEVE performs similarly well in terms of memory usage as all other methods, except for SiFit, which consumes tremendous amounts of memory. The favorable run time and memory performance of SIEVE is also now mentioned in the Discussion (page 17).*

One thing I did not get very much clearly (maybe in real data, or on specific simulated examples), what are the major tree-structure differences we can expect to find employing a prior in SIEVE, that we have against an ML approach - is there any substantial difference in the type of evolutionary story we tell, or in the end is more a statistical difference?

*This is an open question, pertaining more to the differences between frequentist and Bayesian approaches in general, rather than to our particular problem. In principle, here we do not have any a priori expectations for major tree-structure differences.*

*It may happen though that for a particular dataset there will be differences. We now explore this for simulated data and for three real datasets, showing the overall advantage of using SIEVE for evolutionary inference and variant calling (among others, outstanding performance in phylogeny reconstruction, calling double and single mutations, identifying ADOs, capability of identifying mutation events that other methods are not able to account for, favorable run time and memory consumption, robustness to occurence of CNAs). This advantage, however, might be due to several factors that are not directly related to the Bayesian approach, but rather to having a better model of the sequencing data, for example, by modeling ADO, or to differences in implementations, such as differences between RaxML and BEAST 2.*

The paper should also be largely improved on the actual real-data application part. The main criticism is that this FSA assumption seems to be very important for the TNBC example, and the authors say that

"By mapping non-synonymous mutations to the internal branches, we identified different types of mutation events (Methods), including several violations of the ISA, such as back mutations and parallel mutations."

I do not know if I missed that (I did not find it in the Methods), but I think that a clear account of which events are associated to back-mutations and parallel evolution are important, as well as a potential explanation of why that would happen.

*Thank you for this comment. Indeed, our model follows the finite sites assumption (FSA), which is both more general and more plausible than the infinite sites assumption (ISA). Events violating the ISA are expected biologically and probabilistically (10.1038/s41588-021-01005-8, 10.1101/gr.220707.117). It is important to explain that per definition, SIEVE and other models that follow the FSA are well suited to model both cases (when ISA is violated and not). In particular, our model does not enforce violations to the ISA, and consequently, it does not enforce the occurrence of such events like parallel evolution or back mutations. This is now explained in more detail in two new subsections “Summary of model assumptions” and “Summary of evolutionary features accounted for by the model” in Methods (page 34 and 35).*

*For example, we expect that data generated under a phylogenetic model with a very low mutation rate (such as 10-6 used in one set of our simulation scenarios) essentially follows the ISA (i.e., there are very few violations to ISA). In contrast, for high mutation rates (such as 3x10-5 used in another set of our simulation scenarios), we expect the data to violate the ISA. Both these cases are accounted for in our model. Indeed, the performance of SIEVE in both simulation scenarios demonstrates that our model captures both cases well (see Fig. 2). In contrast, for SCIPhI, which follows the ISA, the performance visibly drops for the high mutation rate. This result has already been described in the original manuscript in sections 6*

*“SIEVE accurately estimates tree topology and branch lengths.” (page 6) and “SIEVE accurately calls single and double mutations.” (end page 8, beginning page 9).*

*For real data, the ground truth, in particular about the mutation rates, is unknown and it is impossible to tell a priori whether violations to ISA are expected or not. However, we demonstrate that our model accounts for both cases also on real data. Specifically, for the CRC28 dataset, we find only a single type of mutation event, namely single mutation (0/0 -> 0/1), and SIEVE does not suggest any violations to the ISA. This is now described in “SIEVE inferred a phylogenetic tree and called variants for CRC cells” (page 11).*

*In contrast, as pointed out by the Reviewer, for the TNBC cancer (Fig. 4), our model detects the following diverse types of mutation events (the types of events are encoded by the different colors of the gene names): ● Homozygous coincident double mutation (0/0 -> 1/1) ● Homozygous single mutation addition (0/1 -> 1/1) ● Parallel single mutation (0/0 -> 0/1) ● Single back mutation (0/1 -> 0/0) Notably, the events like back mutations and parallel evolution were much less frequent than the standard single mutations. Indeed, we only found 7 single back mutations and 2 parallel single mutations. These results are now described in more detail in “SIEVE inferred a phylogenetic tree and called variants for TNBC cells” subsection, page 13.*

*Taken together, since per definition FSA does not exclude ISA and is rather its generalization, and given the SIEVE’s results for simulated data, and that SIEVE did not find such events like parallel evolution or back mutations for all real datasets, and that both these events were very rare, we expect these events to recapitulate the real history of the tumor and not be enforced by the model.*

*To improve the clarity regarding the types of mutation events that SIEVE detects, we summarize the underlying genotype transitions and the mutation events that they correspond to in Table 1. In particular, we list the genotype transitions that correspond to back mutations. We also now clarify when parallel evolution is detected (as events that re-occur on independent branches of the tree). The Table 1 and the types of mutation events are described in more detail in page 5 and in an improved subsection “Variant calling, ADO calling, maximum likelihood gene annotation, and mutation event classification” in page 33.*

Moreover, I would like to see how much a tree with ISA would differ from the one with FSA, and in general I would like to see the trees from different methods to be compared rigorously.

*As suggested by the Reviewer, we applied all other models that we considered in the manuscript: SCIPhI, CellPhy, and SiFit to all real datasets and inferred trees using these methods. We then performed pairwise comparisons of the resulting trees, measuring both the branch score distance and the normalized Robinson-Foulds distance.*

*The trees returned by these methods are now featured in new Supplementary Figs 11, 12, 14, 15, 18, 19, 20, 21. The differences are discussed in the main text in page 12 for the CRC28, in page 13 for the TNBC16 dataset, and in page 16 for the CRC48 dataset. 7*

For instance, in one case it is claimed that because the posterior over SIEVE's tree is higher than one on the tree returned by another method, then the SIEVE tree is better. THat is true assuming the bayesian priors in SIEVE make sense -- which I can agree with -- but a more thorough presentation of this difference is important to understand how much actual "evolutionary biology" we miss by using the wrong method.

*Thank you for your comment. The tree that we were referring to was constructed in the original study that generated the TNBC16 dataset (10.1038/nature13600). In that study, the authors used hierarchical clustering of the SNVs to infer the tree. In page 15 we now added an extended description of the additional insights that SIEVE brings about the evolutionary history of a tumor in comparison to hierarchical clustering. Posterior values are only one example of such additional insights.*

Right now - if I am not mistaken - the most sophisticate biological digression by the authors is that cells form clades based on anatomical collection in the CRC case studies. Is there anything else we can discover from this complex phylogenomic methods and their advanced statistical properties? I think this is a key aspect that would make the paper more appealing to the GB readership.

*Thank you for this comment. We have extended the description of the results for the three datasets that we analyzed. We now better introduce the types of mutation events that SIEVE is able to capture (Table 1 in page 5 and in subsection “Variant calling, ADO calling, maximum likelihood gene annotation, and mutation event classification” in page 33) and describe such types of mutation events that were found for each dataset in more detail. We discuss examples of data that seems to follow the ISA (CRC28) and that seems to violate this assumption and displays back mutations or homopleasis (TNBC16). We further identify genes that might have played important roles in the evolutionary histories of these tumors, such as tumor progression and metastasis drivers, or genes that could have affected the mutation rate due to their role in DNA damage and repair pathways. These new insights have been added in page 13 for the CRC28, page 15 for the TNBC16, and page 16 for the CRC48 dataset.*

*Indeed, for evolutionary biologists the tree is an important starting point, enabling various further downstream evolutionary analyses (e.g., see 10.1126/science.aah6266). However, as this is a Method paper, we mostly focus on introducing the methodology behind SIEVE as a computational approach for estimating phylogenetic trees, and on analyzing its accuracy and efficiency, showing that it outperforms other methods in this task.*

- Is the paper of broad interest to others in the field, or of outstanding interest to a broad audience of biologists?

I think the paper is of interest to a subset of the cancer community, namely only to the people carrying out sc-WGS or similar assays to apply this method.

*Although we applied SIEVE to scWGS and scWES data, it is also applicable to targeted sequencing data with a user-specified number of background sites for acquisition bias correction. Furthermore, we showcase SIEVE on cancer data, but the method could also be applied to other scDNA-seq data to trace lineages of healthy cells. We now added these points to the discussion (page 18).*

**Second round of review**

**Reviewer 1**

**Please provide your comments for the author(s) on the revised manuscript.**

I thank the authors for their detailed responses. I find the additions significantly improve the manuscript and address all of my concerns adequately. I have no other questions or revisions to suggest.

**Comments to Author:**

As noted in my review comments, all of my concerns have been addressed sufficiently. I believe this is an innovative and technically strong paper that will be of interest to methods developers in the field and potential users among those generating tumor variation data.

**Reviewer 2**

**Please provide your comments for the author(s) on the revised manuscript.**

I think the authors addressed all of my comments.

**Comments to Author:**

I think the authors addressed all of my comments.
